# Urban scaling laws arise from within-city inequalities

**DOI:** 10.1038/s41562-022-01509-1

**Published:** 2023-01-26

**Authors:** Martin Arvidsson, Niclas Lovsjö, Marc Keuschnigg

**Affiliations:** 1 The Institute for Analytical Sociology, Linköping University, Norrköping, Sweden; 2 Institute of Sociology, Leipzig University, Leipzig, Germany

**Keywords:** Complex networks, Sociology

## Abstract

Theories of urban scaling have demonstrated remarkable predictive accuracy at aggregate levels. However, they have overlooked the stark inequalities that exist within cities. Human networking and productivity exhibit heavy-tailed distributions, with some individuals contributing disproportionately to city totals. Here we use micro-level data from Europe and the United States on interconnectivity, productivity and innovation in cities. We find that the tails of within-city distributions and their growth by city size account for 36–80% of previously reported scaling effects, and 56–87% of the variance in scaling between indicators of varying economic complexity. Providing explanatory depth to these findings, we identify a mechanism—city size-dependent cumulative advantage—that constitutes an important channel through which differences in the size of tails emerge. Our findings demonstrate that urban scaling is in large part a story about inequality in cities, implying that the causal processes underlying the heavier tails in larger cities must be considered in explanations of urban scaling. This result also shows that agglomeration effects benefit urban elites the most, with the majority of city dwellers partially excluded from the socio-economic benefits of growing cities.

## Abstract (web summary)

The authors use large-scale data on urban productivity, innovation and social connectivity, as well as extensive mathematical modelling, and show that power-law urban scaling laws arise out of urban inequalities.

## Main

In recent years, researchers from across disciplines have identified striking and seemingly universal relationships between city size and various urban quantities^1–5^. Cities’ total outputs increase more than proportionately with increases in city size, suggesting that inhabitants of larger cities are, on average, better off economically. This relationship has been described by a power-law function of the form *Y*~*Y*_0_*N*^*β*^, where *Y* represents a city-aggregated socio-economic quantity, *N* is population size, *Y*_0_ is a normalization constant and *β* is a scaling exponent capturing the non-linear change in *Y* as a function of *N*. Estimates of *β* > 1 indicate greater socio-economic output per capita with increasing city size.

To explain such superlinear scaling relations, reference has been made to increasing levels of social interconnectivity in dense urban environments^4,5^. This interpretation meets earlier descriptions of cities as ecosystems of social exchange^6–9^ and, remarkably, simple formalizations of cities as interconnected networks provide predictions that map very well onto empirical observations of superlinear scaling^4,5,10–12^. More recent research has added economic complementarities and the higher industrial complexity found in larger cities to the list of crucial drivers of urban scaling phenomena^13–15^.

However, the main tenet of the scaling paradigm assumes strong levels of homogeneity. It assumes that the residents of a city have roughly equal numbers of network contacts and that the companies in a specific urban industry have similar levels of economic complexity, and thus—as implied by the theory—have approximately equal levels of productivity. Empirical studies have built on this assumption in their use of city sums and means to capture agglomeration effects, as well as in their interpretations, which focus on the ‘average’ resident or firm^5,12,13,16^. Prior research therefore implicitly painted a picture in which scaling effects are driven by a homogeneous shift of the whole city distribution as the population grows larger (see Discussion for further elaboration and Supplementary Note 6 in the Supplementary Information for a detailed review of a recent mathematical framework^17^ that represents the state of the art).

The homogeneity assumption is attractive because it renders mathematical models tractable and empirical analyses straightforward. But—as literature from both the social sciences and complexity research has documented—human networking and productivity show heavy-tailed distributions in which small fractions of extremely well-connected^18,19^ or highly successful individuals^20,21^ contribute large proportions to city totals. Power laws are common in nature and society, present not only as scaling laws between cities but also as extremely skewed distributions within agglomerations^22^. Consequently, sums and means are poor and potentially misleading indicators of the relevant quantities of cities^23–25^.

Acknowledging the extreme skewness of urban indicators implies a discrepancy between observed distributions and the assumptions made by theoretical models, and it suggests an inadequacy of the measures used to test their predictions. A number of questions that go beyond the scope of current theoretical models naturally follow from this. Does within-city tailedness—which we define as the relative contribution of the top ten percentiles in a city, and which therefore also reflects urban inequality—differ systematically by city size? If so, how much of the previously reported superlinear scaling can be attributed to differences in cities’ tails, defined here as individuals or firms in the top (≥90th) percentiles of within-city distributions, as opposed to differences in their mass, which we define as being represented by the typical resident or firm (50th percentile) in a given city. Do phenomena that have heavier tails scale more or less than those with smaller tails? If they do, how much of the variance in scaling exponents across complexity categories (for example, occupations and industries) can be explained by differences in the tails? And, importantly, if within-city tails turn out to be essential to between-city scaling, what mechanisms underlie the emergence of tail differences by city size? These are the questions that we aim to answer in this article.

We use micro-level data from Sweden, Russia and the United States that provide detailed information of within-city distributions of interconnectivity, productivity and innovation. First, we call attention to urban indicators’ heavy tails, particularly in larger cities. Second, we quantify the implications that differences in city tails have for urban scaling. Our findings show that cities’ tails—and, crucially, their growth as cities become larger—disproportionately contribute to superlinear scaling between cities. While we obtain scaling coefficients for city means that are in line with prior results, we find that cities’ tails are responsible for 36–80% of the observed superlinearities across indicators. Additionally, we find that tails explain most of the differences in scaling coefficients between indicators of various levels of complexity. This implies, for example, that once within-city tails have been discarded, average productivity differences by city size are similar for starkly different sectors, such as information technology firms and restaurants. Third, we provide a formal description—in the form of a computational model—of the positive link between the size of tails of within-city distributions and scaling exponents. The model marks out the conditions that give rise to a city size-dependent cumulative advantage mechanism, according to which large cities provide for some people novel opportunities for sustained growth, and it shows how tail differences by city size are brought about at the macro level. The model reproduces our main results, and micro-level data on the earnings trajectories of 1.4 million Swedes confirm the model’s prediction of greater cumulative advantage effects for tail units in larger cities, and thus of their disproportional contribution to superlinear scaling.

These results have ramifications for the dominant mean-field interpretation of urban scaling. In revealing the crucial role of within-city tails, our findings point towards a different understanding of between-city scaling, where agglomeration effects operate on and intensify urban inequality. Our research implies that the causal processes underlying heavier tails in larger cities constitute an indispensable element of urban scaling, and that any theory seeking to explain urban scaling—whether it be through interconnectivity, complexity or other factors—must also explain the emergence of tail differences by city size.

## Results

### Urban indicators are heavy tailed, and more so in larger cities

**Fig. 1:**
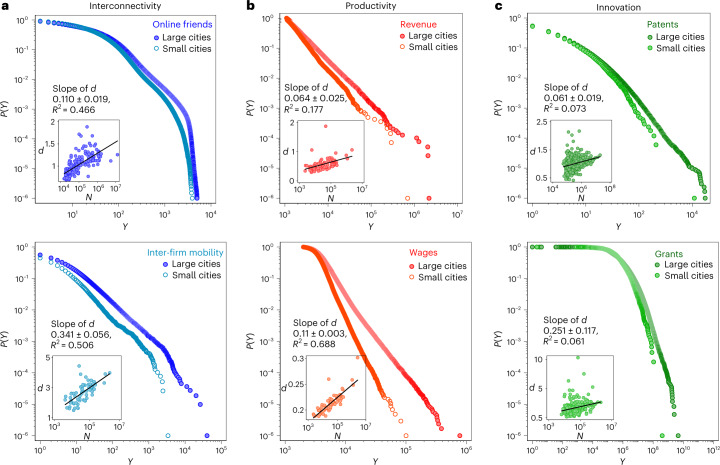
Urban indicators are heavy tailed in cities, and tails increase with city size. We plot the complementary cumulative density function *P*(*Y*) of various urban quantities *Y* in larger (*N* > 1 million) and in smaller cities (*N* < 100,000). **a**, Interconnectivity: the number of online friendships on the Russian social media platform VKontakte in larger and smaller cities, and the degree of companies in employee-mobility networks in Stockholm and Göteborg (>1 million) and in Sweden’s smaller labour market areas (<100,000). **b**, Productivity: company revenue per employee and annual wage in Sweden. **c**, Innovation: the number of patents filed per inventor and the sum of research grants (US dollars) awarded to researchers in larger and smaller US Metropolitan Statistical Areas. Note that the finding of heavier tails in larger cities is independent of bin selection and shows similarly for different intervals. The insets plot *d*, our measure of how strongly city tails dominate urban indicators (see text for a definition), against city size. Regressing *d* on log(*N*), the slope is positive and highly significant (*P* < 0.001, two-sided test using Bonferroni correction for multiple comparisons) for each indicator. Across indicators, the average correlation coefficient between $\log (N)$ and *d* is 0.527 (*P* ≤ 0.003 for each indicator in a two-sided test using Bonferroni correction for multiple comparisons). This indicates that, on average, the size of cities’ tails increases with city size.

Figure 1 shows, in contrast to the common homogeneity assumption of urban scaling theories, that indicators of interconnectivity, productivity and innovation are highly skewed in cities, and that their tailedness increases with city size^26,27^. The insets plot the degree, *d*, to which heavy tails dominate city outputs in cities of different size; for a given city, *d* equals the proportion of an indicator’s sum that is contributed by the top 10% as compared with the bottom 90%; $d= \mathop{\sum }\nolimits_{i}^{N}({y}_{i};y\ge {p}_{90})/\mathop{\sum }\nolimits_{i}^{N}({y}_{i};y < {p}_{90})$, with individuals *i* = 1, 2, ..., *N* and *y* representing individual-level output. We find that, on average across the different indicators, the top 10% within cities account for 50% of city totals, and that *d* is 44% higher in large (>1 million) compared with small cities (<100,000). For (1) interconnectivity, we use two measures: First, the number of online friendships in 177 Russian cities using data from the social media platform VKontakte. Second, we construct inter-firm networks that trace employee mobility between all private companies within each of Sweden’s 70 labour market areas, and we measure each firm’s degree in the constructed networks. These mobility networks transmit economically relevant information between local workplaces^28,29^. (2) We measure productivity using firms’ revenue per employee and the annual gross wages of all full-time workers in Sweden. (3) We approximate urban innovation on the basis of the number of patents filed per inventor and the US dollar sum of grants awarded to researchers in the US Metropolitan Statistical Areas (for details, see Supplementary Note 1).

### The role of city tails for superlinear scaling

**Fig. 2:**
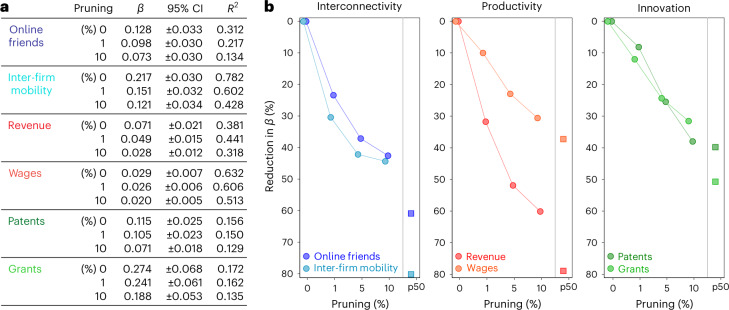
Removing city tails. **a**, We find superlinear scaling coefficients for social interconnectivity, productivity and innovation that are in line with prior research^2–5^ (see also Supplementary Fig. 1). However, the scaling coefficients shrink considerably under differing degrees of tail pruning. **b**, Removing the top 10% of units in each city reduces estimates of *β* by 31–60%, and on average by 41% across the six indicators. Scaling coefficients for city medians (squares), which reflect the mass, are on average 58% lower than those for city means.

To quantify the importance of within-city tails for superlinear urban scaling, we first perform a stepwise exclusion of each city’s tail and re-estimate between-city scaling relations (Fig. 2a). Making full use of the micro-level data, we use per-capita quantities (*Y*/*N*), implying superlinear scaling when *β* > 0 (refs. ^30,31^) (Supplementary Note 2). If superlinear scaling were driven by the mass, rather than the tails, pruning city tails would have only negligible effects on the estimated scaling coefficients. However, removing the most well-connected 10% of social media users in each Russian city and the most well-connected 10% of firms in each Swedish labour market area reduces the exponents for interconnectivity by 43% and 44%, respectively (Fig. 2b). Similarly, pruning the most productive 10% of companies and the top 10% of earners in each Swedish labour market area decreases *β* by 60% and 31%, respectively, and removing the top 10% of inventors and the top 10% of grantees in each Metropolitan Statistical Area reduces the superlinearity of innovativeness by 38% and 32%, respectively.

Second, we completely level any differential tailedness between cities by setting each city’s output to its median value, reflecting a city’s typical social media user, firm, earner, inventor or grantee (squares in Fig. 2b). Compared with the scaling of city means (see the original estimates of *β* without pruning, in Fig. 2a) we find scaling coefficients for city medians that are 36–80% (58% on average) lower across the six indicators (Supplementary Table 1).

Third, we examine the degree to which heavy tails explain deviations from the predictions of superlinear scaling. Deviations from scaling predictions are quantified by the residuals ${\xi }_{c}=\log (\frac{{Y}_{c}}{{N}_{c}}/{Y}_{0}{N}^{\beta })$—known as scale-adjusted metropolitan indicators—and they capture the performance of a certain city *c* = 1, 2, ..., *M* relative to its size for a given urban indicator^32^. We find that, on average, deviations in tailedness explain 34% of the deviations from scaling predictions (Supplementary Fig. 2). This implies that cities that outperform (underperform) on a given urban indicator also tend to have heavier (smaller) tails than would be expected on the basis of their size. As such, within-city tails not only explain a large portion of observed scaling relations, they also account for a considerable part of cities’ deviations from scaling laws, explaining why some cities do better or worse than would be predicted on the basis of their respective sizes.

**Fig. 3:**
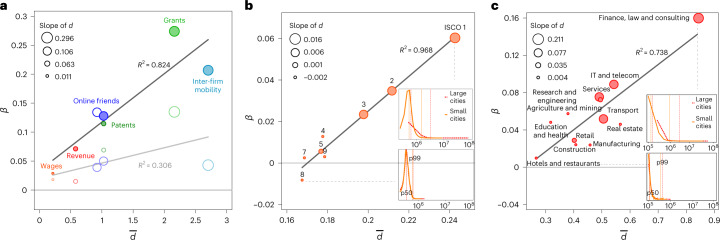
The relationship between inequality and superlinear scaling. **a**, The original estimates of *β* (0% pruning) respond strongly to the indicators’ average within-city skewness $\bar{d}$. The black line approximates this relationship (slope 0.084 ± 0.040, *P* = 0.009 and *R*^2^ = 0.824). At the same time, *β* responds strongly to the increasingly heavy tails as one moves from smaller to larger cities (marker size); a linear fit approximates the relationship between the slope of *d* by city size and *β* at 0.628 ± 0.326, *P* = 0.012 and *R*^2^ = 0.788. Focusing on city medians (empty circles) renders both associations marginal (0.025 ± 0.038, *P* = 0.250, *R*^2^ = 0.306 and 0.165 ± 0.290, *P* = 0.308, *R*^2^ = 0.229, respectively). Expanding the list of measures, we include online friendship networks in Ukrainian cities (empty blue circle: Ukraine; filled blue circle: Russia). **b**, A subgrouping of the wage indicator by differences in occupational complexity (ISCO 1 (9) marking jobs of high (low) prestige) underscores our finding. The ISCO categories closely map onto different degrees of wage skewness in cities. The insets show logged within-city wage distributions (averaged over large (>1 million) and small cities (<100,000)) for categories 1 and 8, respectively; vertical lines mark each distribution’s median and extreme tail (99th percentile). **c**, Repeats a similar analysis for industries of different complexity. Note that, in the case of extremely heavy-tailed distributions within cities (for example, power-law distributions with a scaling parameter *α* < 2), tail differences by city size—and therefore superlinear scaling—may occur completely at random due to a dependency between sample size and the expected value of such distributions^19,57^. The *α* parameters for the six indicators considered here range from 2.1 to 4.4. Confirming the robustness of our results, randomizing units across cities^57^ does not produce scaling exponents that are significantly different from 0 (average *z* score and *P* value across 1,000 randomized populations per indicator and pooled across indicators are 0.795 and 0.381, respectively), and neither are they close to the observed scaling exponents (average *z* score and *P* value across 1,000 randomized populations per indicator and pooled across indicators, in paired *Z*-tests, are 6.788 and *P* < 0.001, respectively) for any of the indicators. We refrain from using *α* to capture the skewness of within-city distributions^59^ because the non-parametric measure *d* proved more robust in smaller cities, where the number of observations for some indicators drops below 50.

To generalize the importance of city tails for superlinear scaling, Fig. 3a uses urban indicators as units of analysis and plots the estimates of *β* for city means against each indicator’s $\bar{d}$ (the mean value of *d*) capturing how strongly heavy tails dominate each indicator across cities. The marker sizes signify each indicator’s slope of *d* (the degree to which its tails grow heavier with city size), which we compute by regressing *d* on the logarithm of city size *N* (see insets in Fig. 1). We find that, for indicators with relatively little within-city skewness, nothing appears to be particularly different in larger cities; their per-capita scaling coefficient is close to 0. Indicators with heavy tails in cities, however, associate with high per-capita scaling. Importantly, the more skewed indicators (with higher levels of $\bar{d}$) also exhibit greater growth in their tails from smaller to larger cities (steeper slope of *d*). As a result of these tail differences, highly skewed indicators have disproportionally more extreme outcomes in larger cities than in smaller ones, and these extreme outcomes contribute crucially to the commonly observed superlinearity of urban scaling relations. Completely removing any tail differences between cities by focusing on city medians (empty circles) renders the association between $\bar{d}$ and *β* insignificant (grey line), and decreases the variance of *β* across indicators by 76%. Consequently, the great majority of the differences in superlinear scaling between urban indicators can be accounted for by differences in the tails.

Previous work has demonstrated that activities of higher complexity show greater scaling coefficients^1,14^. Our results corroborate this link between complexity and steeper scaling coefficients. Importantly, however, our micro-level data also provide a detailed account of previously overlooked heterogeneities within different levels of complexity. We subgroup the wage indicator (Fig. 3b) and firm revenues (Fig. 3c) by differences in economic complexity (for details, see Supplementary Note 1). Figure 3 shows that more complex occupations and industries exhibit heavier tails, and that their tailedness increases with city size. As a consequence, tail differences explain 56% (occupations) and 87% (industries) of the differences in scaling coefficients across complexity levels. For this result, we compare the variance across indicators of different complexity levels when tails are included to the variance when they are pruned (Var(*β*_median_)/Var(*β*_mean_)). This result implies, for example, that most of the difference in scaling that is observed between the finance, law and consulting sector (*β* = 0.159 ± 0.109 and *R*^2^ = 0.154) and the hospitality sector (*β* = 0.010 ± 0.025 and *R*^2^ = 0.012) is due to a minority of very successful finance firms in the largest cities. Once these are removed from the picture, scaling relations become similar for both industries ($\displaystyle{{\beta}_{\mathrm{mean}}^{\mathrm{finance}}-{\beta}_{\mathrm{mean}}^{\mathrm{hospitality}}=0.150\pm 0.106}$ and *P* = 0.007; $\displaystyle{{\beta }_{\mathrm{median}}^{\mathrm{finance}}-{\beta }_{\mathrm{median}}^{\mathrm{hospitality}}=0.026\pm 0.017}$ and *P* = 0.004). The insets illustrate our core argument: while an indicator’s mass (50th percentile) does not differ much between smaller and larger cities, larger cities have heavier tails when the economic activity in question is complex.

### Micro mechanisms behind the inequality–scaling relationship

The central role played by tail differences in explaining superlinear scaling warrants a deeper understanding of their emergence. Here we show that tail differences by city size (Figs. 1 and 2) and the relationship between within-city inequality and scaling exponents (Fig. 3) both logically follow from a joint consideration of three well-established—but so far disparate—strands of research. Bringing together these perspectives on agglomeration dynamics, we propose a mechanism that is supported by micro-level data and that, when simulated, generates the empirical patterns we observe.

The first strand shows how individuals’ productivity depends on the local social environments in which they are embedded, and how this dependency affects agglomeration effects^15,33,34^. Owing to the greater diversity, specialization and matching in larger cities^8,35,36^, scarcer skills tend to concentrate in such cities^13,14,37^. This concentration implies that the skilled and the specialized are more likely to find others whose skills are complemented by their own, allowing for higher levels of productivity in economic activities^15,38,39^. Those whose productivity depends less on whom they interact with—typically the less specialized and the less skilled—do not reap similar returns to complementarity.

The second strand concerns the dynamic benefits of living in larger cities, showing how big-city life facilitates greater learning opportunities and thus, on average, steeper wage trajectories compared with smaller cities^26,40,41^. However, this line of research has yet to consider how learning effects vary as a function of the properties of local social environments in which the inhabitants of a city are embedded (for example, who their peers and colleagues are), and how stochastic selection processes and path dependencies affect individual life courses.

The third strand focuses on the stochastic and path-dependent nature of life courses^42,43^. An individual’s current labour market position, for example, may importantly shape their opportunities in the future^44^. When processes are stochastic and exhibit path dependency, small differences, if accumulated over time, can produce substantial inequality at the collective level^45,46^. It has recently been shown that such dynamics might explain both urban growth and inequality^17^. However, this research does not account for contextual effects, and it disassociates the dynamics that bring about inequality from the processes that give rise to superlinear scaling.

Bringing together these perspectives—the contextual, the temporal and the stochastic—it follows that, for those who are specialized and skilled, (1) large cities provide complementary social environments and novel interaction opportunities that facilitate individual learning and growth, but that (2) such opportunities are accessed to varying degrees by different individuals and (3) that differences accumulate over time due to the stochastic and path-dependent nature of the process. As it pertains to urban scaling and within-city inequality, this implies that, among the specialized and skilled, *β* becomes superlinear because small cities do not provide similar opportunities (and thus inhibit growth); *d*, on the other hand, increases because not everyone can access big-city opportunities to a similar degree, and because opportunities and their effects on individual outcomes accumulate over the life course. By contrast, for the low-skilled and non-specialized, large cities offer few additional interaction opportunities (and thus little or no increases in productivity and inequality). Brought together, the three perspectives give rise to what we call the city size-dependent cumulative advantage mechanism, according to which large cities provide novel but heterogeneously distributed opportunities needed for sustained growth and, at the same time, produce the tail differences by city size that account for a substantial proportion of the overall differences in urban outputs between cities.

**Fig. 4:**
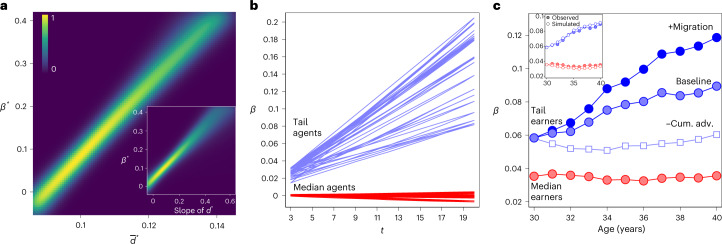
Cumulative advantage drives both tail differences and the positive link between inequality and scaling. **a**, Association between an indicator’s inequality $\bar{d}$ and *β* from simulated data. Across simulation runs, we vary one parameter at a time, considering five different increments that satisfy the criteria $\theta <\!\!\!\!/\!\!\!\!< 0$, $\phi <\!\!\!\!/\!\!\!\!< 0$, *ω* > 0, *τ* ≥ 0 and *δ* > 1 (0.5, 0.75, 1.0, 1.25 and 1.5), while holding the remaining parameters of equations (1) and (2) constant at 1. For each parameter configuration, we generate 1,000 posterior draws of the estimated regression coefficient of β* on ${\bar{d}}^{* }$ (see text for the standardization) to display estimation uncertainty (in total, 21 × 1,000 regression slopes). The colour represents the density over the regression lines extracted from simulated *β* and *d*. The density ranges from 0 (dark blue) to 1 (bright yellow); for relative interpretation, we standardized density values by dividing each by the maximum density. The mean regression coefficient is 8.573 ± 0.209 (95% confidence interval around the mean regression coefficient), *R*^2^ = 0.973. The inset shows the same result for the slope of *d* (0.695 ± 0.023 and *R*^2^ = 0.849). **b**, Emergence of tail differences by city size in simulated data. On the basis of the same simulation runs as in **a**, we trace how the productivity of agents develops, and we estimate the between-city scaling *β* for those who early on (at iteration 2 out of 20) were among the most productive (blue lines) versus the typical agents (red lines). **c**, Using Swedish micro-level data, we trace the wage developments of annual cohorts in each city and, for each year of their early careers, we estimate the between-city scaling *β* among median earners (red markers) and tail earners (blue baseline). Differences in *β* for tail earners and median earners are highly significant at age 30 (*z* = 4.432 and *P* < 0.001, two-sided test using Bonferroni correction for multiple comparisons) and at age 40 (*z* = 7.839 and *P* < 0.001). Zeroing out the size-dependent cumulative advantage (cum. adv.) effect (blue squares) reduces tail differences by 33% (*z* = 3.788 and *P* < 0.001). Once we factor in migration (see text), *β* increases by 33% (*z* = 3.559 and *P* < 0.001). The inset shows that our estimated equivalents to the real-world wage trajectories (empty circles) reproduce the observed scaling exponents (full circles) by cohort age.

We implement an agent-based computational model to demonstrate how the positive link between *d* and *β* can be derived from synthesizing the temporal, contextual and stochastic perspectives. The computational model combines a selection model (equation (1)), specifying how individuals come to interact with particular others, and a learning model (equation (2)), specifying how an individual’s productivity and learning depends on the properties of their interaction partners. We use equations (1) and (2) to simulate dynamic processes of interaction and productivity separately for a number of cities of different sizes. From the simulated city-level data we compute our key quantities of interest, *d* and *β*, on which we base the results presented in Fig. 4a,b. In the following, we outline the key aspects of the simulation model and refer to Supplementary Note 4 for further details, parameter choices and sensitivity analyses. It should be noted that the predictions of our computational model are of a qualitative nature. Our aim is not to reproduce the exact values of our empirical analysis, but rather to reproduce the key features of our results with a simple model under a wide set of very general conditions.

First, we define the probability that an individual (agent) *i* of type *j* interacts with another agent of type *k* in city *c* at time *t* as

1 $${P}_{ijkct}=\frac{{F}_{kc}\left({C}_{jk}^{\theta }+{D}_{ikt-1}^{\phi }\right)}{{\sum }_{l}{F}_{lc}\left({C}_{jl}^{\theta }+{D}_{ilt-1}^{\phi }\right)},$$

where *F*_*kc*_ represents the fraction of type *k* agents present in city *c*, *C*_*jk*_ the complementarity between agents of types *j* and *k*, and *D*_*ikt*−1_ is a distance in actor-type space between the agent type that *i* interacted with at *t* − 1 and the currently considered option *k* (for details, see Supplementary Note 4). *θ* controls how the probability of interaction is affected by complementarity. With rising *θ*, agents increasingly seek out complementary interactions. By contrast, agents randomly sample others when *θ* = 0, and agents avoid complementary interactions when *θ* < 0. *ϕ* governs the degree of path dependency, that is, how probabilities of specific interactions are affected by previous interactions. With rising *ϕ*, agents become more likely to interact with others similar to their past interaction partners. When *ϕ* = 0 (or < 0), past interactions do not affect (or negatively affect) future interactions. For a given timepoint *t*, we use equation (1) to simulate interactions within each city *c* by applying multinomial sampling to each individual’s interaction probability distribution and select one interaction per agent. Importantly, the probabilistic sampling of interactions implies that—in line with the aforementioned literature—similar agents may, out of chance, find themselves in different social environments interacting with different types of others early on, such that *P* subsequently diverges between agents.

Second, we simulate individual-level productivity conditional on the complementarity of realized interactions. For an agent *i*, we let their output *y*_*it*_ update between timepoints as

2 $${y}_{it}={y}_{it-1}\left(1+{\alpha }_{0}\frac{{C}_{it}^{\omega }}{{S}_{i}^{\tau }}\right),$$

where *C*_*it*_ is the complementarity of the realized interaction of agent *i* at time *t* (*C*_*it*_ is short for *C*_*ijkt*_ where agent *i* is of type *j* and they interacted with an agent of type *k* at *t*). *S*_*i*_ is an integer between 1 and 10 qualifying *i*’s specialization rank (1 most specialized, 10 least specialized). Note that we define *i* uniquely across cities and therefore drop the index *c* here to simplify notation. *ω* controls the returns to complementarity, and increases with larger returns. When *ω* = 0 (or < 0), complementarity has no effect (or a negative effect) on agents’ productivity. *τ* specifies how returns to complementarity are moderated by agent specialization, with larger values of *τ* indicating a stronger dependency of returns to complementarity on specialization. When *τ* = 0 (or < 0), returns to complementarity are independent of specialization (or higher for less-specialized agents). The constant *α*_0_ defines the maximum learning rate or, more precisely, the maximum increase in productivity at *t*.

Third, having simulated interactions (equation (1)) and updated the productivity *y*_*it*_ (equation (2)) for all agents *i* in all cities *c* at time *t*, city-level means (${Y}_{ct}=\frac{1}{{N}_{c}}\mathop{\sum }\nolimits_{i}^{{N}_{c}}{y}_{ict}$) and city-level tailedness scores (${d}_{ct}=\mathop{\sum }\nolimits_{i}^{{N}_{c}}({y}_{ict};y\ge {p}_{90})/\mathop{\sum }\nolimits_{i}^{{N}_{c}}({y}_{ict};y < {p}_{90})$) can be calculated, which in turn enables the calculation of our key quantities of interest: *β*_*t*_, $\bar{{d}_{t}}$ and the slope of *d*. We demonstrate that equations (1) and (2) generate distributions of *Y* in cities that reproduce the empirically observed positive association between *d* and *β* under very general conditions. These conditions cover a wide range of plausible behaviours and are all strongly supported by previous research (for a discussion of the model’s scope conditions, see Supplementary Fig. 3):

$\theta <\!\!\!\!/\!\!\!\!< 0$, that is, agents do not strongly avoid complementary interaction environments^47,48^,

$\phi <\!\!\!\!/\!\!\!\!< 0$, that is, agents do not offset path dependence by strongly avoiding previous interaction environments^49,50^,

*ω* > 0, that is, agents yield positive returns to complementarity^15,51^,

*τ* ≥ 0, that is, returns to complementarity are positively moderated by specialization and skill^15,52^,

*δ* > 1, that is, specialized agents disproportionately locate in large cities^14,36^.

In creating the simulation environment, we assume an urban system of 100 cities, with sizes drawn from a Zipf rank-size distribution (we assume the largest city to have a population of 100,000, but this choice does not affect our results). In line with results from past research^36,53^, we assign the fraction *F*_*j*_ of different agent types *j* in the entire urban system based on a log-normal distribution, reflecting much larger fractions of some agent types (the non-specialized) while others (the specialized) have a considerably smaller representation. We calibrate the standard deviation of the log-normal distribution to the Swedish labour market data (Supplementary Note 4). We divide the system-level fractions *F*_*j*_ into city-level fractions *F*_*jc*_ under the assumption of a superlinear concentration of scarcer agent types in larger cities and of a sublinear concentration of common types^13,14^. For simplicity, we define the complementarity *C*_*jk*_ of any two agent types *j* and *k* to be a function of their similarity in overall population fractions *F*_*j*_ and *F*_*k*_. For each agent type *j*, we identify the similarity rank (in terms of population fractions) to each alternative agent type *k*, *R*_*jk*_, such that *R*_*jk*_ = 1 if *j* and *k* have the most similar overall population fractions, *R*_*jk*_ = 2 for the second most similar fractions and *R*_*jk*_ = *K* − 1 for the least similar fractions (where *K* is the number of agent types). Then, we define complementarity as an exponential function of *R*_*jk*_, in which complementarity is decaying rapidly with skill distance. We empirically calibrate the exponential function to match the complementarity concentration found in the Swedish labour market (for details and on robustness to alternative specifications of complementarity, see Supplementary Note 4). At the start of the interaction process, each agent’s productivity is identical and thus independent of city size and agent type such that initially *β* = 0 and *d* = 0.1 for all agent types.

Figure 4a shows the association between inequality and scaling that emerges with parameters set to satisfy the criteria $\theta <\!\!\!\!/\!\!\!\!< 0$, $\phi <\!\!\!\!/\!\!\!\!< 0$, *ω* > 0, *τ* ≥ 0 and *δ* > 1 (for parameter choices and results under violations of scope conditions, see Supplementary Note 4). To visualize the association for different parameter settings (see caption)—which result in *β* and *d* of different magnitudes—we plot standardized versions ${\beta }_{s}^{* }=\frac{{\beta }_{s}}{{\sum }_{l}{\beta }_{l}}$ and ${\bar{d}}_{s}^{* }=\frac{{\bar{d}}_{s}}{{\sum }_{l}{\bar{d}}_{l}}$ per specialization rank *s*, where $\bar{d}$, again, is the average *d* across cities. As predicted—and confirming the empirical results in Fig. 3—we observe a robust positive relationship between urban inequality and superlinear scaling; the mean regression coefficient is 8.573 ± 0.209, with *R*^2^ averaging 0.97. Agent types with greater specialization, as was empirically observed for earners in more prestigious International Standard Classification of Occupations (ISCO) categories and firms in more complex industries, experience both greater *β* and $\bar{d}$, while the opposite holds for less-specialized agents (regressing *β* on specialization rank *S* yields a slope coefficient of −0.0378 ± 0.001 and *R*^2^ = 0.826). Another important empirical pattern, which is closely reproduced by the computational model, is that deviations in tailedness explain a large proportion of the deviations from scaling predictions, the so-called scale-adjusted metropolitan indicators (Supplementary Fig. 11). That is, overperforming (underperforming) cities act as if they were larger (smaller) agglomerations by exhibiting heavier (smaller) tails than would be expected on the basis of their size.

Configurations satisfying the general conditions stated above produce the pattern in Fig. 4a because they give rise to a city size-dependent cumulative advantage effect through which big-city opportunities let tail units excel, leading to within-city distributions becoming heavy tailed. For demonstration, Fig. 4b shows the evolution of *β* conditional on agents’ early productivity, defined as either outstanding (above or equal to the 90th percentile in their respective cities) or typical (40–60th percentile). Agents who from an early stage draw on big-city benefits (≥90th percentile; blue lines) achieve sustained growth; they increasingly distance themselves from both the agents within their own city who are not able to access such opportunities (40–60th percentile) and also from everyone in smaller cities where such opportunities do not exist (≥90th and 40–60th percentiles). By contrast, typical agents in big cities—who fail to draw substantial benefits from novel social environments—yield outputs that are almost identical to those of their counterparts in small cities (constant *β* over time; red lines).

We empirically test the predicted size-dependent cumulative advantage effect, using the life-course structure of our micro-level data and tracing cohorts of 1.4 million Swedish wage earners over time (age 30–40 years). We identify ‘tail earners’ in each city who early in their career had outstanding wages (≥ 90th percentile), or ‘median earners’ in their respective 40–60th percentiles. We trace the wage developments and mobility patterns for all annual cohorts that reached age 30 in 1990–2007 over 10 years. For Fig. 4c, we estimate age-specific scaling coefficients using, as cities’ outputs, the average wage of those workers who earned median or tail wages at age 30. With increasing age, we find stronger growth in the scaling exponent for tail earners than for those classified as median earners early in their careers. In support of the size-dependent cumulative advantage mechanism, those who were initially successful in large cities flourished to a greater extent—thereby distancing themselves from both the typical individual in their own city and the tail individuals in smaller cities (blue baseline)—while the typical individuals in both smaller and larger cities experienced almost identical wage trajectories (constant *β* by age; red line). To quantify the impact of differential cumulative advantage effects on tail differences, we estimate counterfactual wage trajectories under the assumption that tail earners’ wages grow at the same rate as those among the subset of median earners in their respective cities who held similar educational degrees (blue squares; for details, see Supplementary Note 5). We find that, by blocking cumulative advantage effects over 10 years, tail differences reduce by 33% ((0.089–0.060)/0.089).

Considering that tail earners in small cities are incentivized to tap into agglomeration benefits just like their big-city counterparts do, leads to the expectation of selective migration, that is, the relocation of promising workers into larger urban areas. Indeed, we find that those classified as tail earners at age 30 are more than twice as likely to have left a small city (18.9%) compared with a large city (8.7%; *z* = 38.786 and *P* < 0.001) up to age 40, and that those who relocated tended overwhelmingly to move to the largest cities (Supplementary Fig. 13). We take up this previously identified mobility mechanism^27,31,37^ in Fig. 4c, such that the scaling exponent reflects both emergent and selection-based output differences by city size (for implementation, see Supplementary Note 5). The dark-blue line quantifies the impact of migration on cities’ tail differences, increasing *β* by 33% as compared with the observed baseline ((0.119–0.089)/0.089). In terms of promoting tail differences by city size, the disproportionate out migration of the most successful individuals from smaller cities results in a reinforcement process that trims the tails in less populous regions while thickening them in larger cities. Together, the two mechanisms account for approximately half of the observed tail differences ((0.119–0.060)/0.119).

## Discussion

Vilfredo Pareto demonstrated a long time ago that small fractions of society often account for large shares of population totals, giving rise to the widely known ‘80/20 rule’, according to which 20% of people own 80% of the output. This observation remains overlooked by the main tenet of urban scaling theories, which instead assumes that inhabitants within the same cities have similar interconnectivity, productivity and innovativeness^2–5,10–12^. In contrast to this work, we have shown that the outliers identified by Pareto are disproportionately located in larger cities (Fig. 1), and as a result, account not only for a major part of the inequality within cities, but also for the inequality between cities, bringing about patterns of superlinear scaling by city size. We find that differences in cities’ tails—depending on whether one looks at indicators of interconnectivity, productivity or innovation—account for as much as 36–80% of previously reported superlinearities (Fig. 2).

We presented a computational model that provides a formal description of how within-city distributions and scaling exponents are positively linked. Building on the assumption that large cities provide novel opportunities of interaction and learning to individuals with varying degrees, the model predicts city size-dependent cumulative advantage at the micro level and tail differences by city size at the macro level, and it marks out the conditions that reproduce our empirical results (Fig. 4). An analysis of the earnings trajectories of 1.4 million Swedes confirmed the prediction of greater cumulative advantage effects for tail units in larger cities and the transformation of these effects into superlinear scaling.

While reflecting higher-level empirical patterns, the agent-based model we have used surely abstracts away many details and particularities observed in the real world. We have assumed highly stylized agent types and complementarity spaces (for robustness checks and alternative specifications of complementarity, see Supplementary Note 4). We have also left out important aspects of cities, for example, that individuals are nested in organizations and in neighbourhoods, that individuals not only interact within cities but also across cities and that individuals can migrate between cities such that the population size and the composition of cities changes over time. For these reasons, we interpret the predictions generated by the computational model to be of a qualitative nature. Still, the model reproduces many of the empirical features that we observe, and it does so under a very wide range of conditions. This suggests the mechanism instantiated by the model has captured something fundamental. Our implementation of the mechanism in another modelling framework (see below) further supports this interpretation.

The observation that tails systematically grow by city size has implications not only for how we understand any given quantity to change by city size, but also for our understanding of why some quantities scale more than others. Past research has documented scaling relations for a wide spectrum of urban indicators, and theories have attempted to make sense of the variation in magnitude between them. One prominent explanation for this variation is provided by complexity research, which postulates that more complex activities show greater scaling coefficients^1,13,14^. Our work supports this thesis, but also elucidates it further, showing that higher complexity promotes heavier tails, and that it is these tails that explain a large part of the scaling differences between complexity levels (Fig. 3). Additionally, our empirical results show that tails not only explain differences in superlinear scaling by complexity levels, but also make sense of variation in scaling exponents among urban indicators where complexity is not an obvious dimension.

Together, our results indicate that the processes arising from urban density—and from social interconnectivity more generally (Supplementary Fig. 15)—are particularly operative in the tails of urban indicators. Our research implies that the causal processes that explain heavy-tailed distributions in cities constitute a critical element of urban scaling, and that any theory which seeks to explain urban scaling—whether it be through interconnectivity, complexity or other factors—must also explain the emergence of differential tailedness by city size. Demonstrating that it is the units located in the tails of city distributions who bring about the superlinear scaling coefficients reported in earlier work, we call for a shift in focus towards the mechanisms that give rise to heavy-tailed distributions in large cities^15,26,54^.

Robert K. Merton famously observed that, over the course of individuals’ lives, resources tend to diverge in such a way that ’the rich get richer’^42,44^, a process commonly referred to as cumulative advantage. In this article, we found evidence for a strong moderator of cumulative advantage: the size of the relevant social environment. Larger cities provide more opportunities and, as a result, sustain longer cumulative chains. This effect, which we have dubbed the city size-dependent cumulative advantage mechanism, helped to explain approximately one-third of the tail differences that emerge between cities with respect to wages. When simulated, the mechanism also helped to reduce a large proportion of deviations from scaling predictions to deviations in tailedness (Supplementary Fig. 11)—a predictive target that provides further evidence for the empirical relevance of the postulated mechanism. Further, the finding that cumulative advantage is conditioned on the size of a city not only has implications for urban scaling, it also contributes to a broader research agenda, including scholarship on social stratification, that seeks to understand the system-level properties that moderate and influence the character of cumulative advantage effects^55,56^.

Recent research has taken important steps along the lines argued for here. First, empirical methodology has been developed to capture within-city variation in scaling^23–25^ and to ensure the robust estimation of scaling exponents in the light of heavy-tailed urban indicators^57^. Second, a recently proposed theoretical framework^17^ has acknowledged heterogeneity and inequality in urbanization processes. Still, that framework remains predicated on a traditional type of urban scaling theory with mass shifts at its core, and where the process underlying inequality and the process underlying scaling are disassociated from one another. By incorporating the size-dependent cumulative advantage mechanism identified here, however, these processes become interlinked and the predictions can be brought into accordance with our empirical observations (Supplementary Note 6), demonstrating the generalizability and explanatory power of the mechanism we proposed here.

From a policy perspective, our analyses underscore that urbanization is no panacea against social inequality. Agglomeration effects are instead particularly beneficial to the city elites that dominate urban hierarchies. Their networks and their affluence depends, more than others, on the local social environments that the largest cities provide. At the same time, the higher-than-expected outputs of larger cities critically depend on the tail outcomes of these successful few. Ignoring this dependency, policy makers risk overestimating the stability of urban growth, particularly in the light of the high spatial mobility among urban elites, their movement to ‘where the money is’ and their dependence on specific industries and on these industries’ long-term growth trajectories.

Zooming out to the system level, the size-dependent cumulative advantage mechanism operating on the micro level accumulates into a city-level rich-get-richer process, where the largest cities benefit from strong path dependencies in the composition of individuals and firms, and from the attraction of further tail units from beyond the city^31^. Dominant positions in the urban hierarchy thus give an advantage to larger cities^58^. This path dependency also limits the self-similarity of growth paths of cities, implying that cities that at a certain time have very different sizes are not self-similar ‘scaled versions of one another’^2^, predicted to experience similar growth paths. Instead, they are cities with very different relative status and thus provide the social environments that the successful few strive on to very different degrees.

With respect to urban inequality, our findings draw attention to the partial exclusion of a majority of city dwellers from the socio-economic benefits of growing cities. Their lifestyle, different than among the urban elite, benefits less from geographical location. When accounting for the cost of living in larger cities, many big-city dwellers will in fact be worse off as compared with similar people living in smaller places. In light of the extreme inequalities that exist within urban populations, our results stand in stark contrast to a mean-field interpretation of superlinear urban scaling that is derived from, and dependent on, homogeneity assumptions, and they raise questions about the sustainability of urbanization against the backdrop of rising inequality in cities.

### Reporting summary

Further information on research design is available in the Nature Portfolio Reporting Summary linked to this article.

## Supplementary information

Supplementary Information Supplementary Figs. 1–15 and Table 1.

Reporting Summary

## Data Availability

The aggregated micro-level data that support the findings of this study are available for download at the Open Science Framework (DOI: 10.17605/osf.io/uhsmz). We collected the online networking data for Russia and Ukraine through the VKontakte API, the data on US patents are from the US Patent and Trademark Office and on research grants from Dimensions. The Swedish micro-level data come from administrative and tax records and can therefore not be shared; access may be requested from Statistics Sweden. We use publicly available data on city demarcations and population sizes from the Russian Federal State Statistics Service, the State Statistics Committee of Ukraine, Statistics Sweden and the United States Census Bureau.

## Code availability

The code used in this study is available at the Open Science Framework (DOI: 10.17605/osf.io/uhsmz). We used R (Version 4.0.5) for data analysis and simulations, Python (Version 3.6.4) for InfoMap-based network clustering, and Stata (Version 15) for visualizations. We used R (Version 4.0.5) to collect the online networking data and the data on patents and research grants. The code for these data collections is available upon request.
