# Supplementary material for: Urban scaling laws arise from within-city inequalities
Source: Nat Hum Behav. 2023 Jan 26;7(3):365–74. doi: 10.1038/s41562-022-01509-1 (PMC10038794; doi:10.1038/s41562-022-01509-1)
Supplement: Supplementary file 1 — Supplementary Figs. 1–15 and Table 1. [file 41562_2022_1509_MOESM1_ESM.pdf]

nature human behaviour

Article

https://doi.org/10.1038/s41562-022-01509-1

Urban scaling laws arise from within-city inequalities

In the format provided by the authors and unedited

Note 1. Data and measures

Online networking data: We measure the number of online friends of 40 million users on VKontakte (`https://vk.com/dev/openapi`), a popular Russian social media platform resembling Facebook in style and functions, in Russian cities in 2018. We exclude cities with coverage rates below 1/3 of their population as well as all users for who residential information is lacking. The remaining user data cover 177 cities with an average coverage rate of 47% ± 2% and population sizes (number of users) ranging from 15,700 (6,700) to 12.4 million (6.7 million). The average number of friends is 60 ± 0.06. For the scaling analysis of city sums (Supplementary Fig. 1), we follow ref. 5 and extrapolate the average number of friends for each city to the city's full population by dividing the observed sum by the fraction of the population registered on VKontakte. This correction adjusts for local variations in platform popularity that are unrelated to interconnectivity differences. City demarcations and population sizes are from Russia's Federal State Statistics Service (`https://eng.gks.ru`). At the time of data collection, VKontakte was also widely used in Ukraine, allowing us to replicate our finding with regard to this indicator in a second urban system (Fig. 3A). 87 Ukrainian cities—with population sizes (numbers of users) ranging from 16,800 (7,800) to 2.9 million (2 million)—exceed the 1/3-coverage threshold (average coverage: 51% ± 7%). These data cover 10.5 million users with on average 52 ± 0.08 online friends. City demarcations and population sizes are from the State Statistics Committee of Ukraine (`http://ukrstat.gov.ua`).

Company and wage data: Gross annual revenues and wages, reported directly by tax authorities, are available from Swedish population registers (`https://scb.se/en`). Both indicators are measured in 2017 in hundreds of Swedish krona (roughly equivalent to units of 11 current US$). The registers contain information on every organization and every employee located in any of the country's 70 labor market areas, Sweden's functional demarcation of urban areas[1], ranging from 2,272 to 2.2 million in population size. For the revenue analysis, we use annual revenue per employee and exclude secondary businesses (those with an average annual revenue-per-employee below 100 thousand Swedish krona, approx. 9.1 thousand US$). The wage indicator focuses on the 3,462,150 fully employed earners, excluding informal wages that fall below the minimum wages set on the basis of industrial agreements in Sweden. The wage indicator enables a subgrouping of earners based on the International Classification of Occupations (ISCO-88) codes. In Fig. 3B, we categorize occupations into the 9 major ISCO groups (ranging from managers to ordinary workers). This grouping reflects differences in the complexity of tasks that are also highly correlated with occupational prestige. The linear correlation between this grouping and the International Socio-Economic Index (a standard measure of occupational prestige[2] ranging from 16 [low prestige] to 90 [high prestige]) is $r = -0.837$ ($p < 0.001$). We exclude ISCO-category 6 (skilled agricultural and fishery workers) from the analysis due to its low level of representation in cities. The registers, further, enable us to trace employees' year-by-year mobility between the country's numerous private firms, from kebab shops to multinational corporations. We compute professionals' inter-firm mobility during the

period 2015–17 separately within each labor market area, and we consider a total of 259,721 unique firms (on average 188,197 per year). In Fig. 3C, we categorize companies into 12 industries of various levels of economic complexity (ranging from "Hotels and Restaurants" to "Finance, Law, and Consulting'), based on the Swedish Standard Industrial Classification (SNI 2007) codes.

Patents and grants data: We collected information on all new patents registered during the period 1976–2019 by the US Patent and Trademark Office (`https://www.patentsview.org`) and connected each patent-holder's geographical information to their respective Metropolitan Statistical Area (MSA; `https://census.gov`). The data cover 8.3 million patents from 1.8 million US inventors in 379 MSAs. Research grants include both public and private monetary funding awarded to researchers in the US during the period 1960–2019, as reported by the research data platform Dimensions (`https://www.dimensions.ai`). We compute each awardee's total funding in (inflation-adjusted) US$ and, based on the awardee's main affiliation, assign it to the respective awardee's MSA. We consider 892,000 grants awarded to 363,000 researchers in 218 MSAs.

Note 2. Estimation of β for city sums, medians, and tails

Following standard practice[3] when estimating the scaling exponent β for city-aggregated totals (i.e., the sum of a particular quantity for each city), we reformulate the power-law function $Y_c(N) \sim Y_0 N_c^{\beta}$—where Y is an aggregate quantity of city $c = 1, 2, ..., M$, N is population size, and Y_0 is a common baseline—as a linearized model

$$\log Y_c = \log Y_0 + \beta \log N_c + \epsilon_c,$$

in which c extends over cities and ϵ_c is a normally distributed error with zero mean. We estimate β using linear ordinary least squares regression minimizing $\sum_{c=1}^{M} (\log Y_0 N_c^{\beta} - \log Y_c)^2$—the sum of cities' squared distances to a linear best fit function relating city size to urban output. The linear function's slope provides an estimate of β, and superlinear scaling implies $\beta > 1$. Supplementary Fig. 1 shows the results of a scaling analysis based on the indicators' city sums.

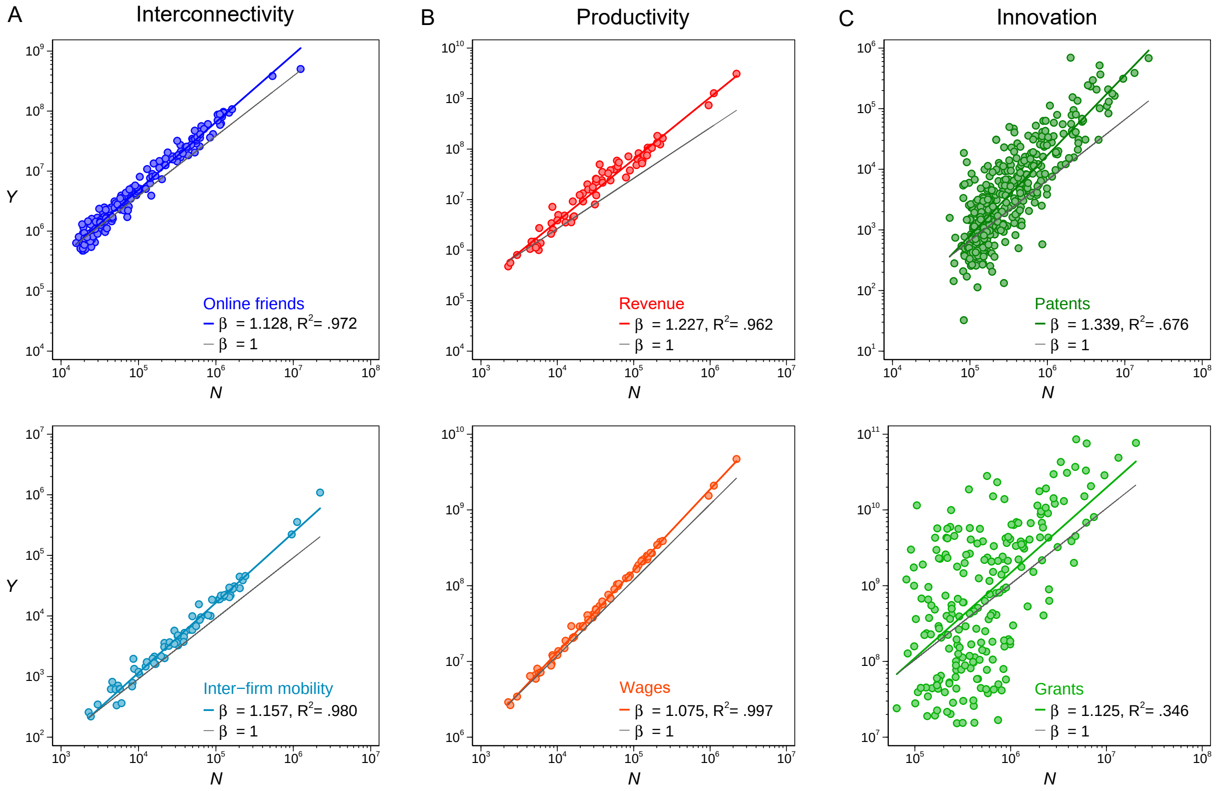

Supplementary Figure 1. Scaling of city sums. Our data confirm the previously reported superlinear scaling relations for city sums[3–6]. (**A**) Interconnectivity: Total number of online friends [$\beta = 1.128 \pm 0.033$ (95% confidence interval around the coefficient obtained from linear regression), $R^2 = 0.973$] and total company degree in inter-firm mobility networks [$\beta = 1.157 \pm 0.051, R^2 = 0.980$]. (**B**) Productivity: Total annual revenue per employee [$\beta = 1.227 \pm 0.044, R^2 = 0.962$] and total annual wage [$\beta = 1.075 \pm 0.014, R^2 = 0.997$]. (**C**) Innovation: Total number of patents [$\beta = 1.339 \pm 0.086, R^2 = 0.676$] and total sum of research grants [$\beta = 1.125 \pm 0.180, R^2 = 0.346$]. Gray lines indicate proportional scaling relations ($\beta = 1$).

Y		β	95% CI	R^2
Online friends	Median	0.050	±0.034	0.063
	Mean	0.128	±0.033	0.312
	99th pct	0.231	±0.048	0.453
Inter-firm mobility	Median	0.043	±0.041	0.104
	Mean	0.217	±0.029	0.782
	99th pct	0.225	±0.051	0.535
Revenue	Median	0.015	±0.011	0.122
	Mean	0.071	±0.021	0.381
	99th pct	0.196	±0.055	0.425
Wages	Median	0.018	±0.005	0.458
	Mean	0.029	±0.007	0.632
	99th pct	0.096	±0.015	0.786
Patents	Median	0.069	±0.029	0.073
	Mean	0.115	±0.025	0.156
	99th pct	0.196	±0.039	0.173
Grants	Median	0.135	±0.054	0.076
	Mean	0.274	±0.068	0.172
	99th pct	0.338	±0.088	0.162

Supplementary Table 1. Per-capita scaling coefficients in the mass and in the tail of urban indicators. Scaling coefficients for city medians are, on average, 58% lower than for city means. The extreme tails (99th percentile) generate scaling coefficients that are on average about two times greater than those for city means, and more than four times greater than those for city medians.

Taking full advantage of the micro-level data, we shift to the use of per-capita quantities in our main analysis, substituting each city's mean quantity for its sum

$$\log \frac{Y_c}{N_c} = \log Y_0 + \beta \log N_c + \epsilon_c.$$

This implies superlinear scaling at $\beta > 0$ and a considerable decrease in the "apparent fit"[7] of the scaling model. Supplementary Table 1 summarizes the per-capita scaling coefficients for city medians, city means, and the 99th percentile in each city. It is worth noting that differences $\beta_{sum} - \beta_{mean} \neq 1$ can occur, reflecting the dependency of the extensive β_{sum} on the different numbers of units measured in different cities. Whereas β_{mean} permits contrasting the average productivity of, say, occupation X in cities of various sizes, β_{sum} reflects differences both in average productivity and in the relative numbers of X across various cities. The per-capita perspective thus enables a finer-grained analysis of urban scaling.

Note 3. Urban inequality and cities' deviations from scaling laws

To further investigate the importance of within-city tails for the aggregate outputs of cities, we quantify the extent to which *deviations* in within-city tailedness explain *deviations* in urban outputs from the predictions of scaling laws. We measure cities' deviations from scaling predictions by their residuals $\xi_c^{(Y)} = \log(\frac{Y_c}{N_c}/Y_0 N^{\beta^{(Y)}})$, which are known as "scale-adjusted metropolitan indicators" (SAMIs)[8]. For a given urban indicator, these residuals capture the performance of a certain city relative to its size. Similarly, we can compute a measure of "scale-adjusted tailedness" (SATs), $\xi_c^{(d)} = \log(d_c/d_0 N^{\beta^{(d)}})$, that captures deviations in within-city skewness relative to expectations based on a city's size (see also the insets in Fig. 1).

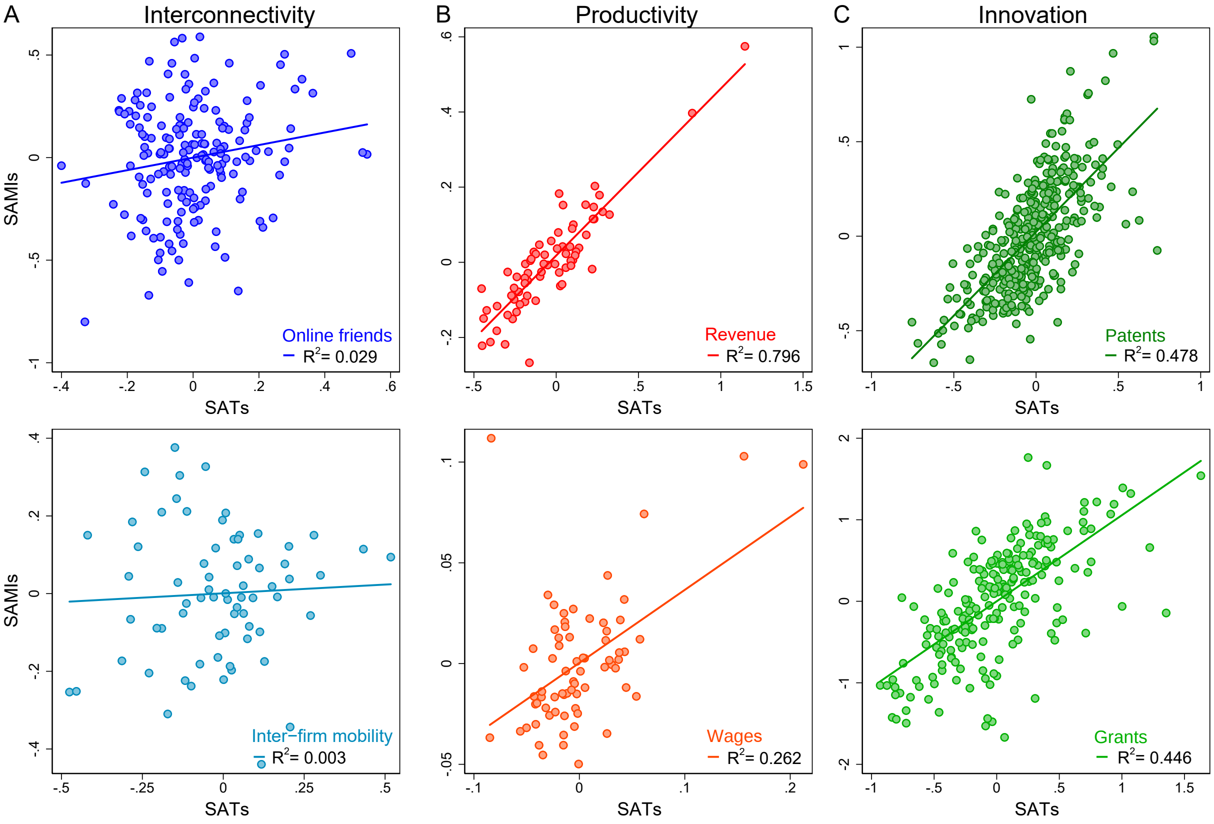

Supplementary Figure 2. Deviations in city tailedness and deviations from scaling predictions of urban output. (**A**) Interconnectivity: Linear association between scale-adjusted tailedness (SATs) and scale-adjusted metropolitan indicators (SAMIs) for the mean number of online friends [slope $= 0.306 \pm 0.269$ (95% confidence interval around the coefficient obtained from linear regression), $R^2 = 0.029$] and the mean company degree in inter-firm mobility networks [$0.045 \pm 0.216, R^2 = 0.003$]. (**B**) Productivity: Mean annual revenue per employee [$0.444 \pm 0.046, R^2 = 0.796$] and total annual wage [$0.362 \pm 0.222, R^2 = 0.262$]. (**C**) Innovation: Average number of patents per inventor [$0.886 \pm 0.116, R^2 = 0.478$] and average sum of research grants [$1.056 \pm 0.177, R^2 = 0.446$].

Supplementary Fig. 2 shows that, on average across indicators, deviations in tailedness explain 34% of the deviations from scaling predictions. Hence, cities with heavier (smaller) than expected tails also tend to overperform (underperform) in relation to what would be

expected on the basis of their size. As regards variation between indicators, interestingly, this association is almost non-existent for indicators of interconnectivity, for which we find the greatest average reduction in superlinear scaling when tail differences are removed altogether (Fig. 2). Further research will be needed to explore this heterogeneity between the different urban quantities.

Note 4. Details of the simulation, scope conditions, and robustness analyses

This section provides an overview of the agent-based computational model (see pseudo code below), summarizes details on variable construction, parameter choices, scope conditions, sensitivity analyses, and an additional analysis that demonstrates how the simulated city-size dependent cumulative advantage mechanism reproduces the positive association between cities' deviations in tailedness (SATs) and their deviations from scaling laws (SAMIs) that we reported in Supplementary Note 3.

Construction of the variables for complementarity C and path-dependency D. The simulation assumes K agent types ($j = 1, 2, ...K$), each having an overall population fraction $F_j = \frac{N_j}{N}$, where N_j is the number of j-type agents, and $N = \sum_j^K N_j$ the total number of agents. We assume that F_j is inversely linked to the level of specialization and skill of agent type j. The distribution that we assume for F—and its dependence on city size—resembles what we observe empirically: When we classify employees in the Swedish labor market along specialization levels as proxied by years of education (as in ref. 15), the inequality (generalized entropy) in representation across specialization levels is ≈ 0.2. In the simulation, we draw F from a lognormal distribution with a standard deviation parameter σ set such that the generalized entropy across specialization levels (S) approximates 0.2 ($\sigma = 0.7$). Further, the parameter δ, the degree to which specialized agents disproportionately locate in large cities, regulates how F varies by city size (for details and demonstration of robustness, see "Parameter settings" further down). For each agent type j we can create a ranking $R_{jk} \in \{1, .., K-1\}$ based on how similar j is to another agent type k in terms of skill and specialization: $R_{jk} = 1$ implies maximum skill-level similarity of k to j, and $R_{jk} = K-1$ implies minimum skill-level similarity. R is the basis for constructing both C and D.

We define the complementarity C_{jk} between two agents j and k as an exponential function of R_{jk}: $C_{jk} = e^{-\eta R_{jk}}$, with η controlling the rate at which complementarity is decaying with the skill distance in R. That is, more similarly skilled agents (e.g., same level of education) are assumed to be more complementary compared to less similarly skilled agents (e.g., different level of education). This assumption is strongly supported by empirical data. In the Swedish labor market, the average abundance of a given specialization level (again, proxied by years of education) predicts the specialization level with $R^2 = 0.84$ (the lower the abundance, the higher the specialization level). Similarity in specialization level, in turn, predicts complementarity with $R^2 = 0.70$ (the higher the similarity in specialization level, the higher the complementarity). Further, this simple definition enables the encoding of two important empirical features of complementarity: (i) the highly skilled and specialized can more easily find complementary others in big cities than in small cities, and (ii) the mass of complementarity is concentrated on a relatively narrow range of agents types. The parameter η regulates the degree of this concentration. In the simulations presented in Fig. 4, we set $\eta = 0.07$. This value reproduces the level of concentration we observe in the Swedish labor market data (generalized entropy index of pairwise complementarity scores ≈ 0.40). Robustness analyses presented in Supplementary Fig. 5 show insensitivity of results to this parameter choice.

Pseudo code for the agent-based computational model

Create environment:

Assign population fraction F_j to each agent type $j \in \{1, ..., K\}$

Assign complementarity score C_{jk} for all agent-type pairs j,k as a function of F_j and F_k

Assign city size N_c to each city $c \in \{1, ..., M\}$ following a Zipf rank distribution

Assign city fractions F_{jc} for each agent type j in each city c such that $\sum_j^K F_{jc} = 1$, $\sum_c^M F_{jc} = 1$, and that F_{jc} is dependent on N_c and F_j

Initialization:

Set $y_{ict=0} = 100 \; \forall \; i \in \{1, ..., N\}$ and $c \in \{1, ..., M\}$

Begin simulation:

for $c \in 1 : M$ **do**

    for $t \in 1 : T$ **do**

        for $i \in 1 : N_c$ **do**

            Calculate interaction probability distribution P_{ict} using Eq. 1

            Apply multinomial sampling to P_{ict} to select one interaction partner for agent i

            Measure complementarity C_{it} of the realized interaction

            Update productivity y_{ict} for agent i using Eq. 2

        end for

        Calculate city c's average productivity at t: $Y_{ct} = \frac{1}{N_c} \sum_i^{N_c} y_{ict}$

        Calculate city c's tailedness score at t: $d_{ct} = \sum_i^{N_c}(y_{ict}; y{\geq}p_{90}) / \sum_i^{N_c}(y_{ict}; y{<}p_{90})$

    end for

end for

Compute scaling $\beta_t \; \forall \; t \in \{1, ..., T\}$ via estimating $\log \frac{Y_{ct}}{N_{ct}} = \log Y_0 + \beta_t \log N_{ct} + \epsilon_{ct}$

Compute average tailedness score across cities $\bar{d}_t$

Compute the slope of d (η_1) by city size via estimating $d_{ct} = \eta_0 + \eta_1 \log(N_c) + \epsilon_{ct}$

We define path-dependency D_{ijkl} as an exponential function of the skill-level distance in R between the agent type the focal agent i interacted with at $t-1$ (l) and the agent type under consideration at time t (k): $D_{ijkl} = e^{-\lambda|R_{jk}-R_{jl}|}$, where R_{jk} is the similarity rank between the focal agent's type (j) and the agent type under consideration (k), R_{jl} is the similarity rank between j and the agent type that i interacted with at $t-1$ (l), and where λ controls the rate at which the density decays around R_{jl}. Thus, D reflects the distance in skill and specialization between the agent type under consideration and the agent type of the past time point, and λ regulates the concentration of what is considered to be similar agent types. In the simulation results presented in Fig. 4, we set $\lambda = \eta = 0.07$ such that the similarity of agents is tied to similarity in complementary. This implies that, if path-dependency is important and agent i interacted with a highly complementary agent in $t-1$, i is inclined to interact with highly complementary agents also in t. That said, our results are insensitive to the exact specification of λ (see robustness analyses in Supplementary Fig. 5).

Parameter settings. Fig. 4 demonstrates the computational model's ability to reproduce the key features of our empirical findings under conditions that satisfy the postulated criteria ($\theta \not\ll 0$, $\phi \not\ll 0$, $\omega > 0$, $\tau \geq 0$, and $\delta > 1$). The simulations consider for each of the

parameters in Eqs. 1 and 2 a baseline value of 1. Over simulation runs, we vary parameter values by two increments on each side of the baseline (0.5, 0.75, 1.0, 1.25, 1.5). The parameter ranges cover behavior on both ends of the empirically plausible spectrum. Below, we relate these parameter ranges to behaviors exhibited in the Stockholm labor market, and further down we discuss scope conditions.

θ controls how complementarity affects the probability of interaction. The baseline value of 1 implies that agents select interaction partners proportional to their complementarity score. In the Swedish labor market data, individuals select interactions (i.e., select into firms) approximately proportional to their complementarity scores: the average complementarity for observed interactions (coworker relationships) is 0.58, while it is 0.56 for sampling proportionally based on complementarity returns (the standardized difference between the two means is < 0.1). Then, $\theta = 0.5$ and $\theta = 1.5$ correspond to sampling interactions in which the link between interaction probability and complementarity scores is reduced (increased) by 50%, respectively.

ϕ controls the degree of path dependency. The baseline value of 1 implies that agents select interaction partners proportional to the (exponent) of the similarity distance R to the agent type that they interacted with at the previous time point. The resulting autocorrelation corresponds well with empirical observations. Under the assumption that $\lambda = \eta$ (see above), $\phi = 1$ results in an autocorrelation of 0.55 between C_{it} and C_{it-1} for simulated interactions. In the Swedish labor market data, the corresponding estimate of autocorrelation is 0.59. Then, $\phi = 0.5$ and $\phi = 1.5$ correspond to the sampling of interactions that have 50% less autocorrelation (autocorrelation coefficient: 0.30) and a 30% higher autocorrelation (autocorrelation coefficient: 0.70), respectively.

ω controls returns to complementarity. The baseline value of 1 implies linear returns to complementarity. In the Swedish data, returns to complementarity are approximately linear. For this empirical result, we compare model fit and marginal predictions between a model with only linear terms and one with third-order polynomials, and we find the model fit close to identical and model predictions statistically indistinguishable. We computed complementarity as in ref. 15 as a function of co-occurrence of specializations in workplaces, controlling for substitutability (measured by how correlated two agent types' shares are in different occupations). Then, $\omega = 0.5$ and $\omega = 1.5$ correspond to decreasing (increasing) marginal returns between complementarity and productivity. Setting $\omega = 0.5$ reduces the difference in returns to complementarity between the 80th percentile and the 20th percentile of the complementarity distribution by a factor of 3. Setting $\omega = 1.5$ increases this difference by a factor of 3.

τ controls how returns to complementarity are moderated by specialization. The baseline value of 1 implies that returns to complementarity are proportional to the degree of specialization, i.e., if the returns to complementarity is x for agents with the highest degree of specialization 1, then the returns equal $x/10$ for agents with the lowest degree of specialization 10, a 10-fold difference. In the case of the Swedish labor market, previous research[9] has reported (and our data corroborate) differences in returns to complementarity between individuals *with* and *without* university education that match

this gradient well: the increase in wages associated with moving from the 10th to the 90th percentile in the complementarity distribution is approximately 8 times larger for those with university education compared to those without. Then, $\tau = 0.5$ and $\tau = 1.5$ correspond to changing the returns-to-complementarity ratio between the most specialized and the least specialized from 10 to 3 and from 10 to 32, respectively.

δ controls the disproportional location of specialized agents in larger cities. The parameter represents the scaling exponent of the number of specialized agents in a city dependent on its size. The baseline value for δ differs from the other parameters. Rather than being set to 1, it is set to 1.25 (note the scope condition $\delta > 1$) implying that, as the size of a city doubles, the number of specialized agents increases by 125%. Compensating for the above proportional increase in the number of specialized agents by city size, the fraction of less specialized agents decreases by city size, and thus exhibits sublinear scaling. We consider this parameter range for δ: 1.15, 1.20, 1.25, 1.30, 1.35. In the Swedish labor market data, the scaling coefficient for the number of specialized individuals (approximated by university degree) is 1.23. Differentiating by the length of study, the corresponding estimates are 1.17 (1.28) for those with less (more) than 3 years of university education, and 1.37 for those with a doctoral degree.

Scope conditions of the predicted d–β association. Fig. 4 demonstrated that a positive link emerges between d and β when the conditions $\theta \not\ll 0$, $\phi \not\ll 0$, $\omega > 0$, $\tau \geq 0$, and $\delta > 1$ are met. Here, we consider violations of these scope conditions. Supplementary Fig. 3 shows how the d–β association changes as we vary one parameter at a time, holding the remaining parameters of Eq. 1 and 2 constant at 1. For each simulation, we ran regressions of the simulation output β_S on $\bar{d}_S$, where $S \in \{1, ..., 10\}$ corresponds to the ten specialization levels considered in the simulation. Each data point in a panel represents one regression coefficient for a particular configuration of θ, ϕ, ω, τ, δ.

θ: When $\theta < 0$, agents seek interactions of low complementarity, and as a result, the greater opportunities for complementary interactions offered by big cities are left unused, implying that neither superlinear scaling nor inequality will emerge (panel A).

ϕ: When $\phi = 0$, agents sample interactions without consideration of past interactions. Multiplicative growth theory[10,11] explains why inequality still emerges, both within and across specialization categories: When growth rates in a stochastic process accumulate over time, as is the case here, normally distributed growth rates translate into lognormal outputs. When $\phi \ll 0$, agents strongly avoid past (complementary) interaction environments and the d–β association breaks down (panel B).

ω: When $\omega = 0$, complementarity does not influence agents' output, output is independent of city composition, and neither superlinear scaling nor inequality emerges. When $\omega < 0$, returns to complementarity flip such that productivity and learning decrease with complementarity and the more specialized agent types encounter a sublinear scaling regime. This implies a negative relationship between inequality and scaling (panel C).

τ: When $\tau = 0$, all specialization ranks $S \in \{1, \ldots, 10\}$ yield similar returns to complementarity, but inequality and scaling can still be positively linked, because of the concentration of the specialized in larger cities. When $\tau < 0$, complementarity returns decrease with specialization, and the link between β and d breaks down (panel D).

δ: When $\delta = 1$, fractions of agent-types in cities are independent of city size, making agents, on average, equally likely to find complementary others in both smaller and larger cities. When $\delta < 1$, specialized agents concentrate in smaller cities, and a negative d–β association follows (panel E).

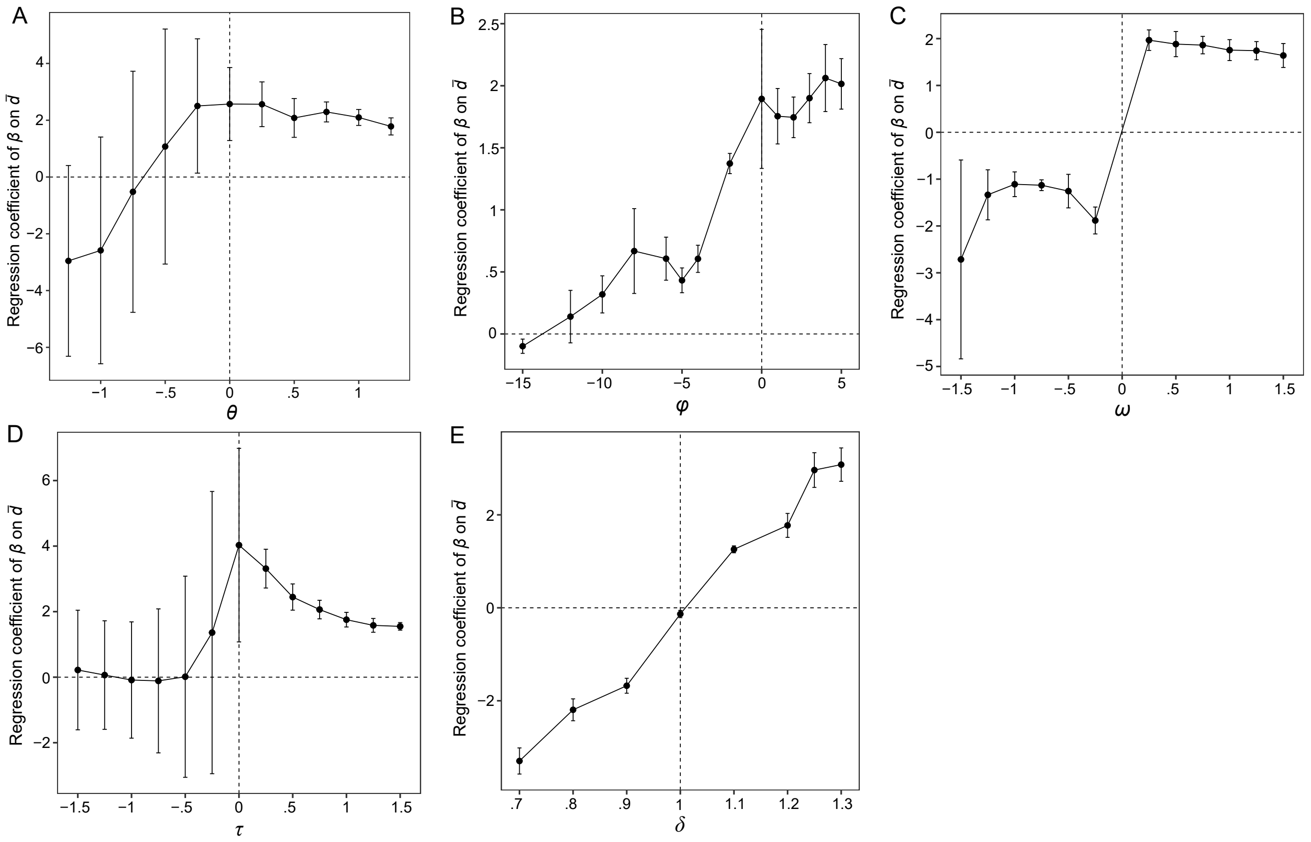

Supplementary Figure 3. Testing the scope conditions of the computational model. The panels show how the association of β and $\bar{d}$ collapses when the conditions $\theta \not\ll 0$, $\phi \not\ll 0$, $\omega > 0$, $\tau \geq 0$, and $\delta > 1$ are violated. We vary one parameter at a time, holding all other parameters of Eq. 1 and 2 constant at 1, and we report the regression coefficient of β_S on $\bar{d_S}$ on the Y-axis, where $S \in \{1, ..., 10\}$ correspond to the different specialization levels. Hence, each data point in the plot represents one regression coefficient for a particular configuration of θ, ϕ, ω, τ, δ. Because we use 10 specialization levels, $N = 10$ for each regression. Error bands represent 99% confidence intervals around each coefficient obtained from the estimated linear regressions.

Robustness analyses. In creating the simulation model, a number of choices were made concerning setup properties and background parameters that are less substantially anchored. Here, we demonstrate robustness to these choices. In addition, we report robustness to alternative specifications of complementarity.

Number of iterations. Fig. 4 and Supplementary Fig. 3 present simulation results after 20 iterations. Our results are insensitive to this choice. Although the absolute values of Y, d, and β continue to change with the number of iterations in this out-of-equilibrium model, the β–d relationship remains stable with increased number of iterations. Supplementary Fig. 4 replicates Supplementary Fig. 3 under varying number of iterations (30, 40, 50). Further, Z tests of differences in correlation between β–d for different numbers of iterations are insignificant for all configurations (average p-value=0.81).

Rate parameters of exponential functions. On the basis of empirical calibration, we set $\lambda = \eta = 0.07$. This specification reproduces the level of concentration observed in empirical complementarity distributions (generalized entropy index ≈ 0.4 and the top decile accounting for $\approx 30\%$ of total complementarity). Again, results are insensitive to this choice. Supplementary Fig. 5 replicates Supplementary Fig. 3 using values for λ and θ that correspond to concentration levels where the top decile accounts for 20% ($\lambda = \eta = 0.04$), 40% ($\lambda = \eta = 0.10$), and 50% ($\lambda = \eta = 0.14$), respectively.

Alternative model specifications. Some aspects of our model specification were left unmotivated in the main text, such as the additive relationship between C and D. The qualitative nature of our results is robust to alternative specifications. For illustration, Supplementary Fig. 6 presents results under an alternative specification of Eq. 1, substituting the additive relationship between complementarity and path-dependency by a multiplicative relationship:

$$P_{ijkct} = \frac{F_{kc} C^{\theta}_{jk} D^{\phi}_{ikt-1}}{\sum_l F_{lc} C^{\theta}_{jl} D^{\phi}_{ikt-1}},$$

City sizes. For the results presented in Fig. 4 and Supplementary Fig. 3, we set the maximum size of a city to 100 thousand, and then used a Zipf rank distribution to assign the size for the remaining cities. The choice of 100 thousand for the largest city has no particular theoretical motivation. Rather, it was motivated on practical grounds: it is large enough for smaller groups of specialized agents to be present in meaningful numbers, while not being too large in terms of computational cost. Our results are highly robust to alternative city size distributions. Supplementary Fig. 7 replicates Supplementary Fig. 3 for simulations using 25K, 50K, and 250K as the maximum city size.

Distribution of agent type population fractions F. As stated above, we use a lognormal distribution for F such that some agent types are considerable more abundant than others. We calibrate the degree of skewness of the lognormal distribution such that the inequality in abundance between different specialization levels S approximates that of the Swedish labor market. Setting the standard deviation of the lognormal, σ, to 0.7 results in an inequality that approximates the empirically observed (generalized entropy = 0.2). Our results are robust to the selection of σ. Supplementary Fig. 8 replicates the scope conditions as reported in Supplementary Fig. 3 for simulations using $\sigma \in \{0.3, 0.5, 0.9, 1.1, 1.3\}$.

Definition of complementarity C. As stated above, we use a definition of complementarity that builds on similarity in specialization level. While there is a strong empirical relationship between the degree of specialization and complementarity, there is no perfect mapping. In reality, educational tracks have qualitative aspects that make some pairs of specializations less complementarity even though they come on similar levels of specialization. This may be especially prevalent at the upper end of the specialization spectrum where subsets of similarly skilled or specialized people might be highly complementary, while others (also similarly skilled or specialized) might be much less complementary. The definition used for our main results does not account for this possibility. To demonstrate robustness to alternative specifications of complementarity, we consider modifications to its original definition. In a first set of simulations, we do one of the following:

1. assume a constant (average) complementarity for focal agent i's 20% most similarly specialized agents,
2. reverse the complementarity order of the 20% most similarly specialized agents.

We apply (1) and (2) first to all agent types, and then only to the two rarest agent types ($S \in \{1, 2\}$). Supplementary Fig. 9 replicates Supplementary Fig. 3 for simulations using these modified definitions of complementarity.

In a second set of simulations, we introduce as an additional dimension of complementarity a *field* of specialization independent of the *level* of specialization. This enables two agents to have an identical specialization level but still have low complementarity. And it enables some agents of different specialization level to be complementary because they share the field of specialization. More specifically, we

3. randomly assign each agent type j to 1 of 2 (or 1 of 3) field categories. If a pair of agent types j,k does not belong to the same field, we reduce their original complementarity (determined by similarity in specialization level) by 50%. For pairs belonging to the same field, their original complementarity is instead increased by 50%.

In one condition of (3), we add the field dimension to all 10 agent types; in a second condition, we add it only to the 2 rarest agent types. Supplementary Fig. 10 replicates Supplementary Fig. 3 for simulations using this second set of modified definitions of complementarity.

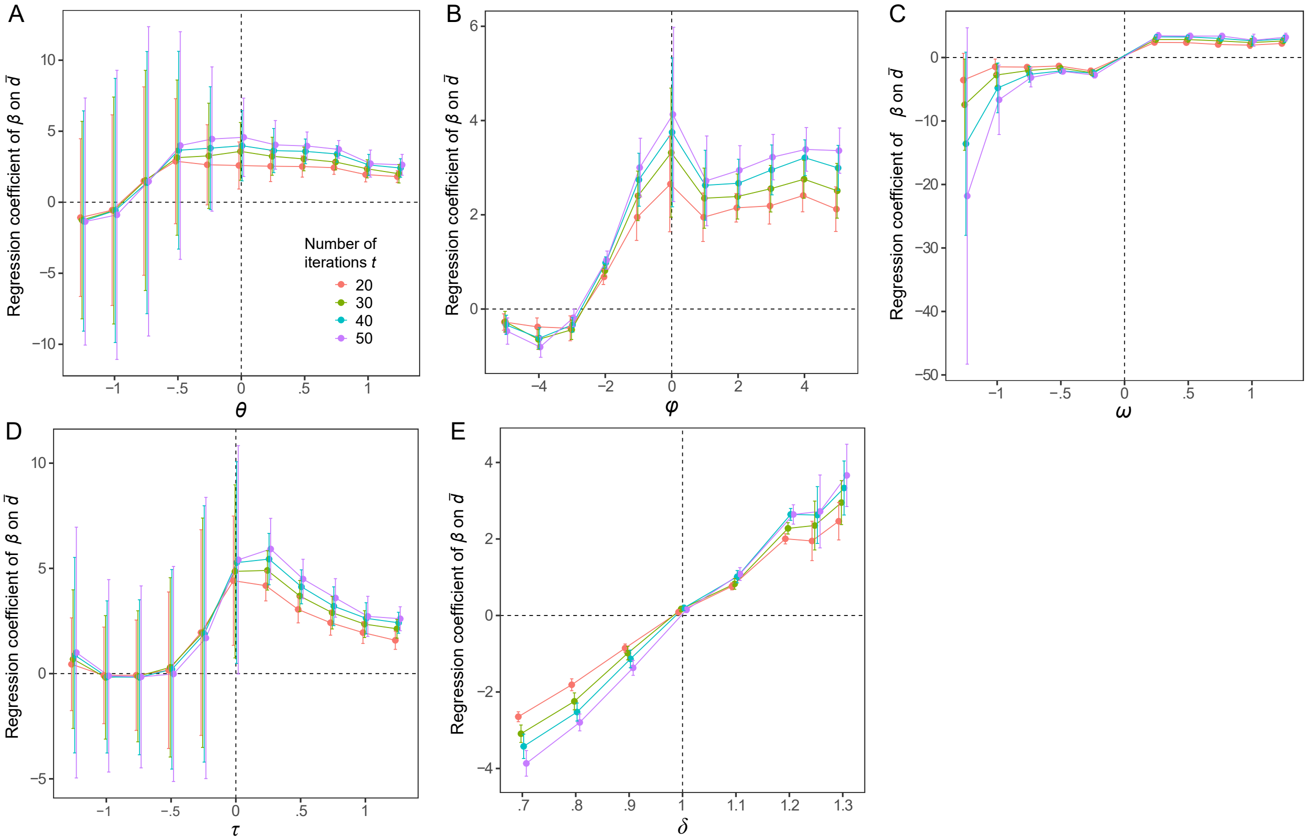

Supplementary Figure 4. Insensitivity to the number of iterations. The panels replicate Supplementary Fig. 3 at 30, 40, and 50 iterations of the simulation. As in Supplementary Fig. 3, the panels show how the association of β and $\bar{d}$ collapses when the conditions $\theta \not\ll 0$, $\phi \not\ll 0$, $\omega > 0$, $\tau \geq 0$, and $\delta > 1$ are violated. We, again, vary one parameter at a time, holding all other parameters of Eq. 1 and 2 constant at 1, and report the regression coefficient of β_S on $\bar{d}_S$ on the Y-axis, where $S \in \{1, ..., 10\}$ correspond to the different specialization levels such that $N = 10$ for each regression. Error bands represent 99% confidence intervals around each coefficient obtained from the estimated linear regressions.

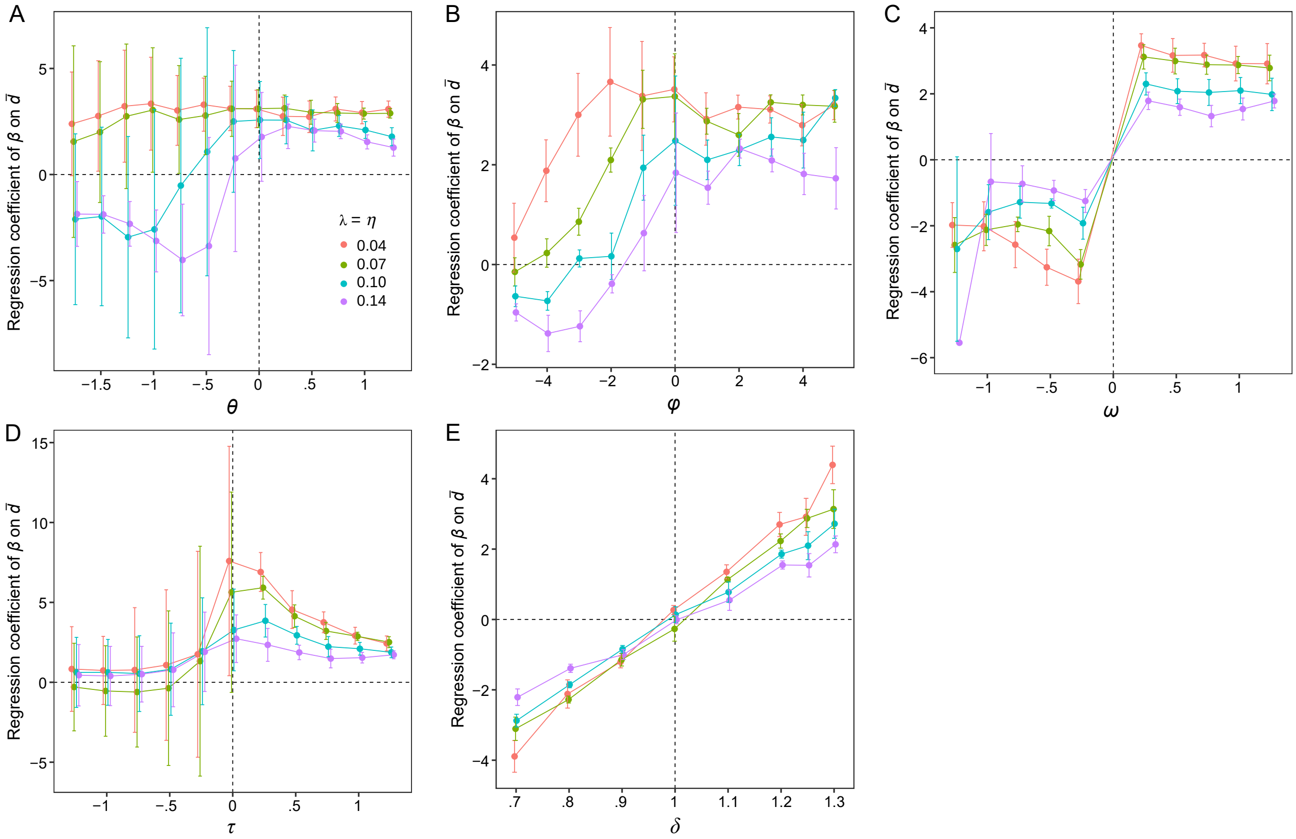

Supplementary Figure 5. Insensitivity to the rate parameters of exponential functions. The panels replicate Supplementary Fig. 3 for different values of λ and θ that correspond to concentration levels where the top decile accounts for 20% ($\lambda = \eta = 0.04$), 40% ($\lambda = \eta = 0.10$), and 50% ($\lambda = \eta = 0.14$). We, again, vary one parameter at a time, holding all other parameters of Eq. 1 and 2 constant at 1, and report the regression coefficient of β_S on $\bar{d}_S$ on the Y-axis, where $S \in \{1, ..., 10\}$ correspond to the different specialization levels such that $N = 10$ for each regression. Error bands represent 99% confidence intervals around each coefficient obtained from the estimated linear regressions.

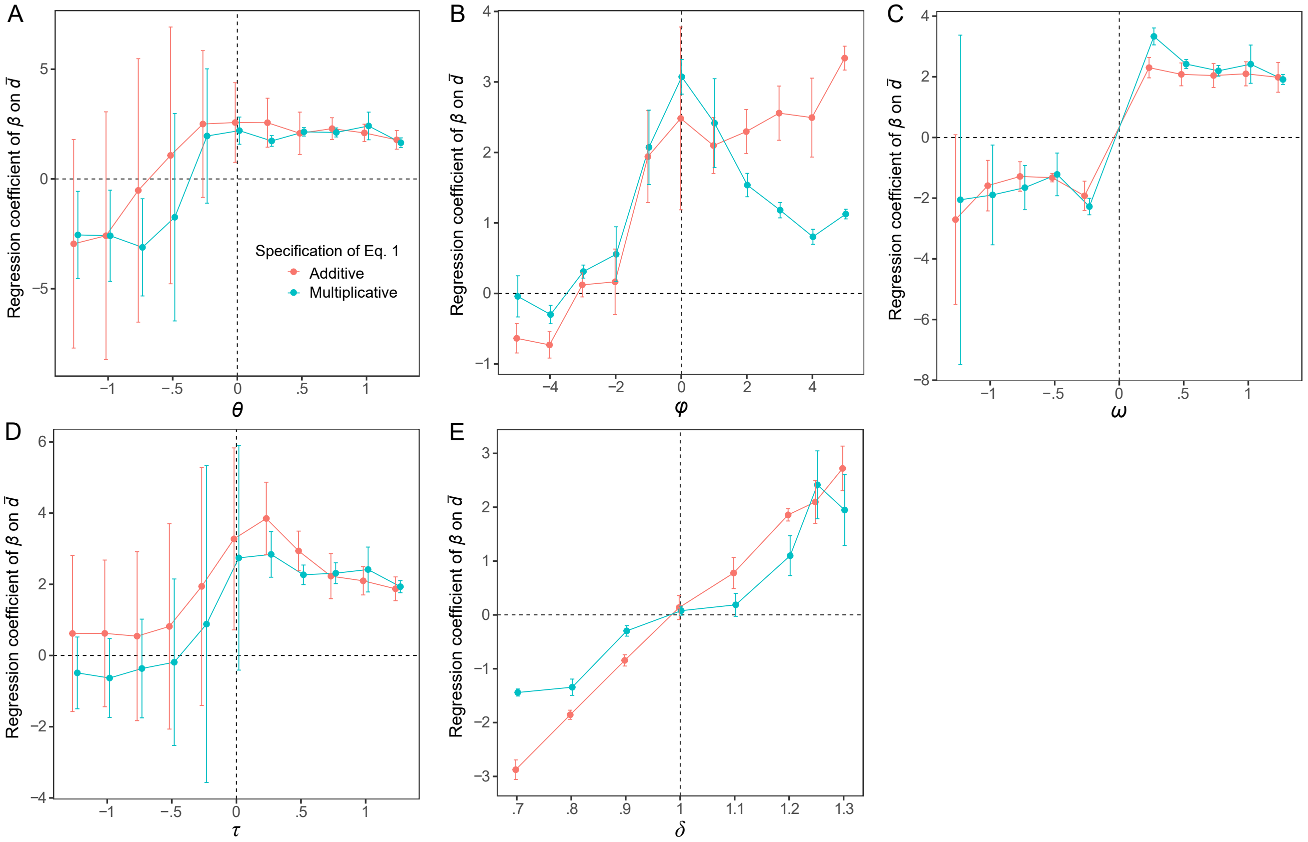

Supplementary Figure 6. Insensitivity to alternative specifications of Eq. 1. The panels replicate Supplementary Fig. 3 also when the additive relationship between complementarity and path-dependency is replaced by a multiplicative relationship. We, again, vary one parameter at a time, holding all other parameters of Eq. 1 and 2 constant at 1, and report the regression coefficient of β_S on $\bar{d_S}$ on the Y-axis, where $S \in \{1, ..., 10\}$ correspond to the different specialization levels such that $N = 10$ for each regression. Error bands represent 99% confidence intervals around each coefficient obtained from the estimated linear regressions.

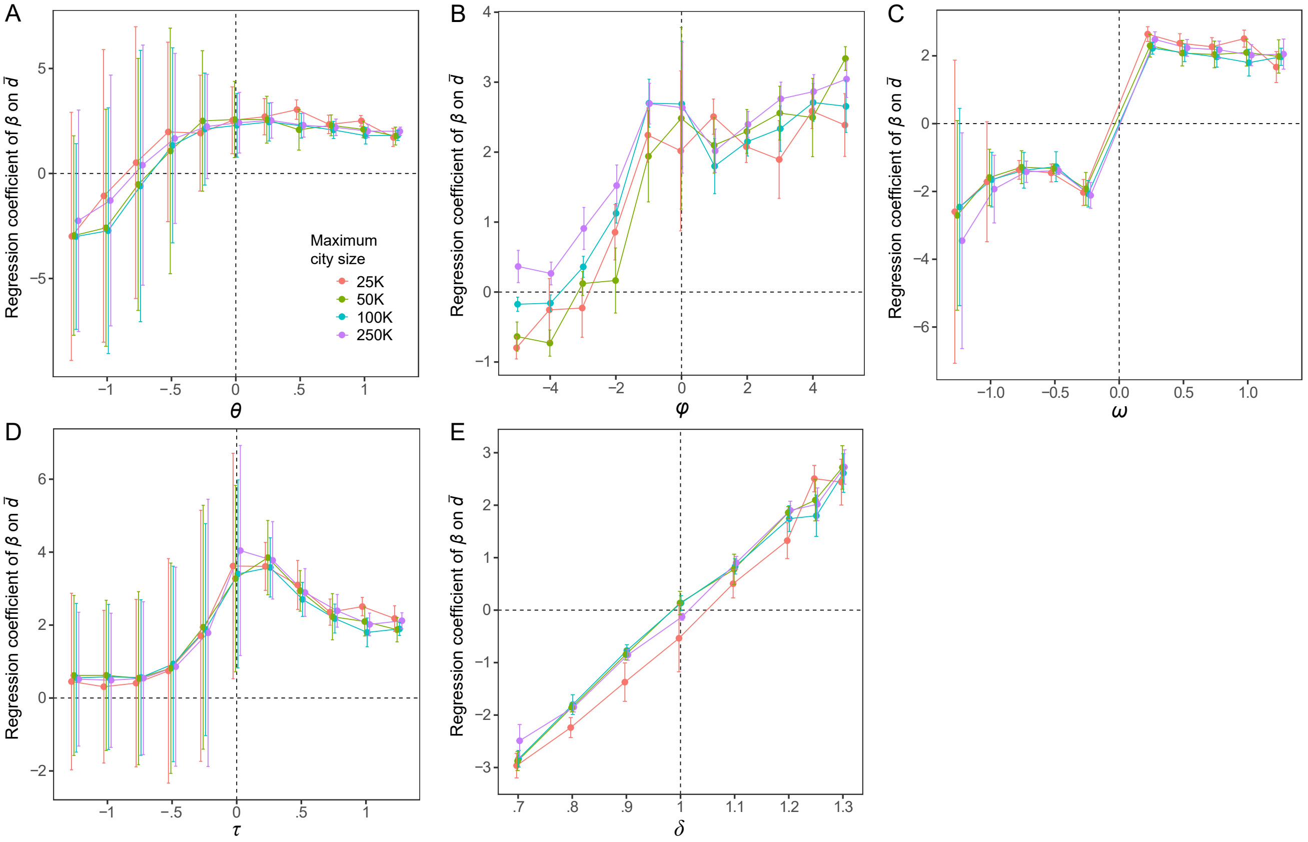

Supplementary Figure 7. Insensitivity to the size of simulated cities. The panels replicate Supplementary Fig. 3 for maximum city sizes of 25K, 50K, and 250K. We, again, vary one parameter at a time, holding all other parameters of Eq. 1 and 2 constant at 1, and report the regression coefficient of β_S on $\bar{d}_S$ on the Y-axis, where $S \in \{1, ..., 10\}$ correspond to the different specialization levels such that $N = 10$ for each regression. Error bands represent 99% confidence intervals around each coefficient obtained from the estimated linear regressions.

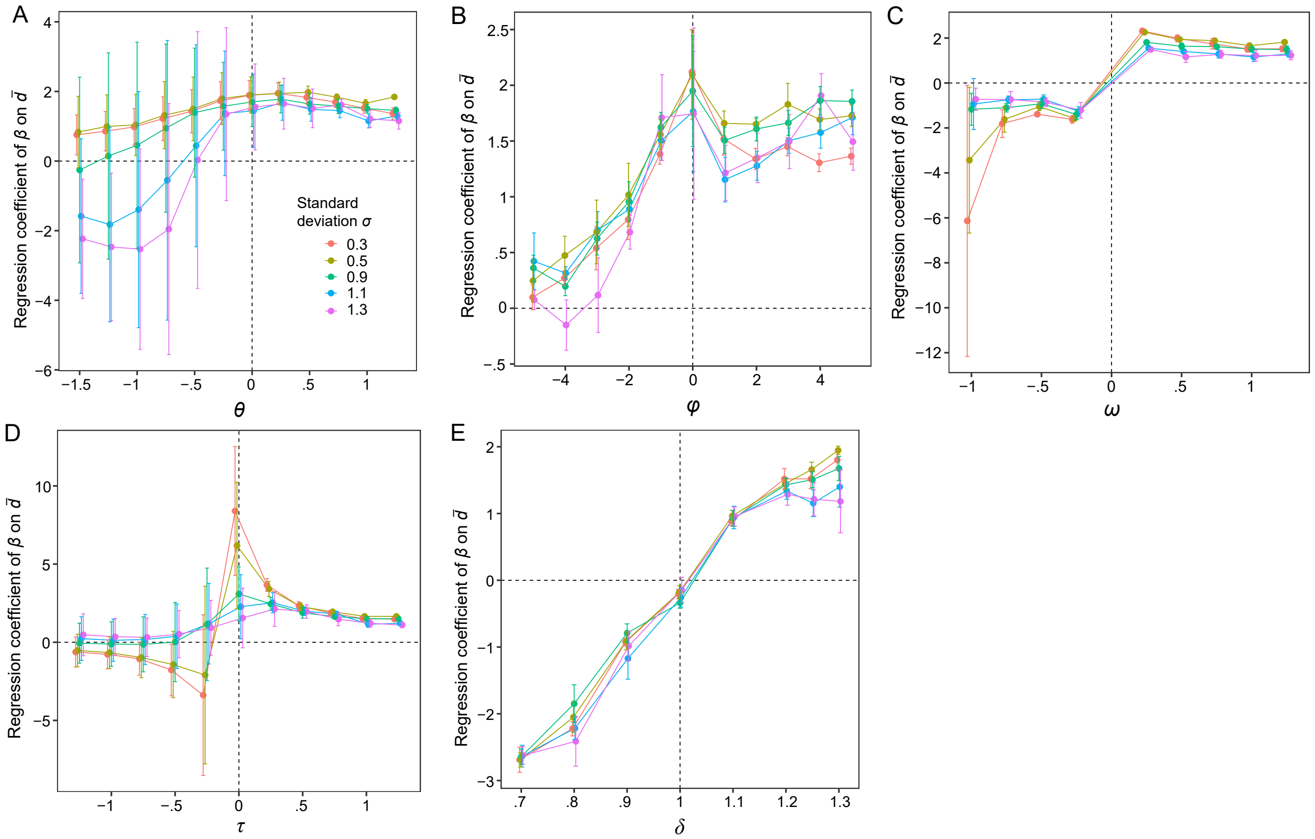

Supplementary Figure 8. Insensitivity to the distribution of agent type fractions. The panels replicate Supplementary Fig. 3 for different degrees of skewness of the lognormal distribution governing the system-wide inequality in agent types' population fractions. We vary the standard deviation of the lognormal $\sigma \in \{0.3, 0.5, 0.9, 1.1, 1.3\}$. We, again, vary one parameter at a time, holding all other parameters of Eq. 1 and 2 constant at 1, and report the regression coefficient of β_S on $\bar{d}_S$ on the Y-axis, where $S \in \{1, ..., 10\}$ correspond to the different specialization levels such that $N = 10$ for each regression. Error bands represent 99% confidence intervals around each coefficient obtained from the estimated linear regressions.

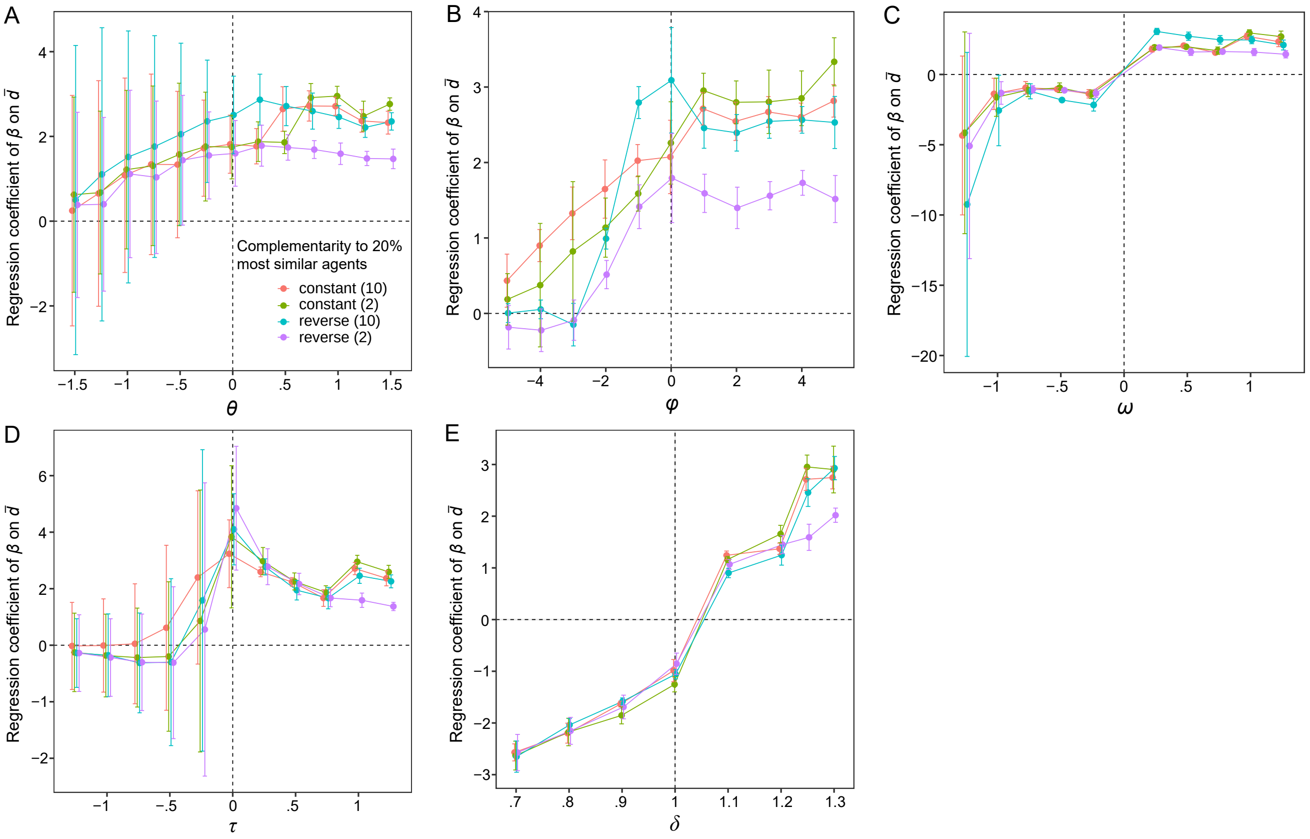

Supplementary Figure 9. Insensitivity to the definition of complementarity I. The panels replicate Supplementary Fig. 3 using the modified definitions of complementarity (1) and (2); see text. We apply modifications to all 10 agent types, and to the 2 rarest agent types. We, again, vary one parameter at a time, holding all other parameters of Eq. 1 and 2 constant at 1, and report the regression coefficient of β_S on $\bar{d}_S$ on the Y-axis, where $S \in \{1, ..., 10\}$ correspond to the different specialization levels such that $N = 10$ for each regression. Error bands represent 99% confidence intervals around each coefficient obtained from the estimated linear regressions.

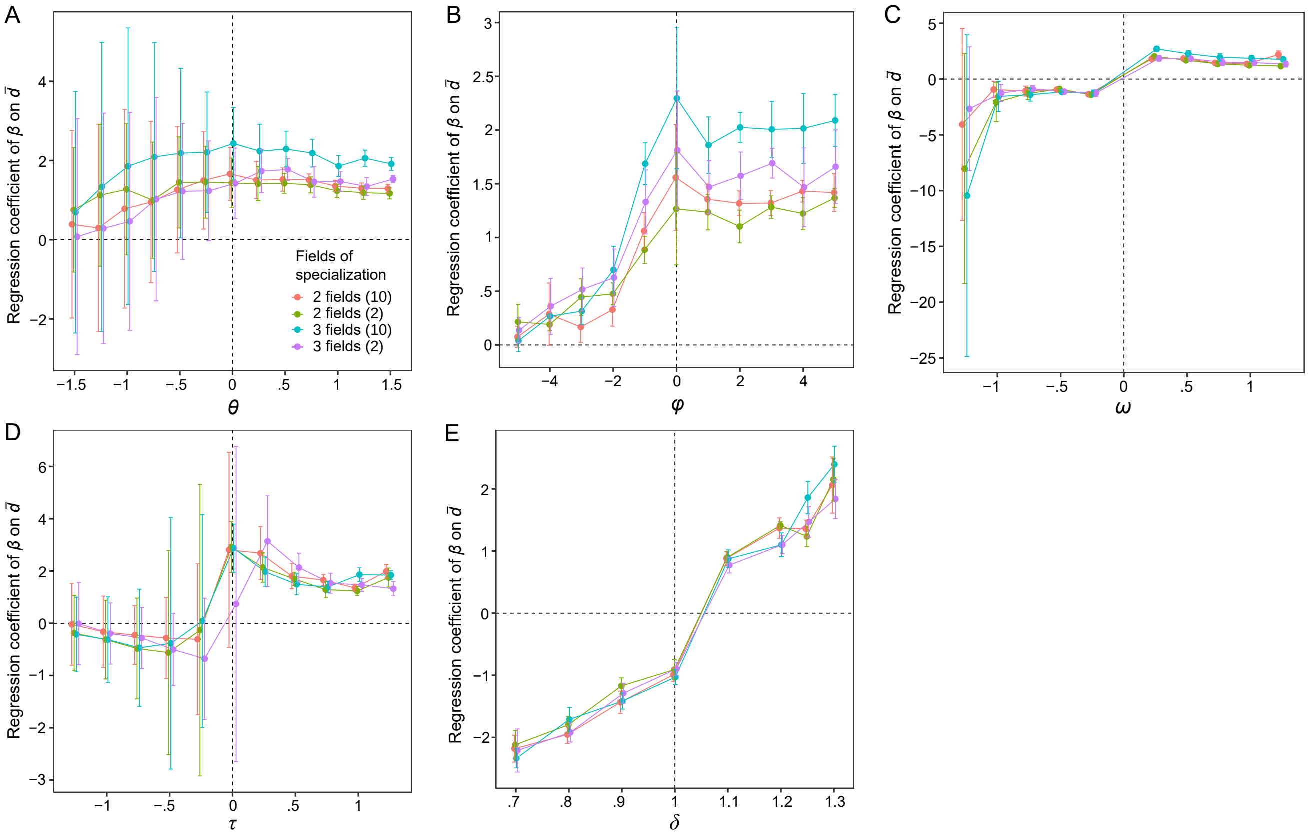

Supplementary Figure 10. Insensitivity to the definition of complementarity II. The panels replicate Supplementary Fig. 3 introducing a second dimension of complementarity (see modification (3) in the text). We apply modifications to all 10 agent types, and to the 2 rarest agent types. We, again, vary one parameter at a time, holding all other parameters of Eq. 1 and 2 constant at 1, and report the regression coefficient of β_S on $\bar{d}_S$ on the Y-axis, where $S \in \{1, ..., 10\}$ correspond to the different specialization levels such that $N = 10$ for each regression. Error bands represent 99% confidence intervals around each coefficient obtained from the estimated linear regressions.

Association between simulated SATs and SAMIs. When simulated, the city-size dependent cumulative advantage mechanism not only brings about tail differences and a positive association between d and β. The mechanism also reproduces the positive association between scale-adjusted tailedness (SATs) and scale-adjusted metropolitan indicators (SAMIs) that we reported in Supplementary Fig. 2. On average, across the simulations presented in Fig. 4, SATs explain 66% of the variation in SAMIs. That is, the overperformance (or underperformance) of a particular city relative to its size can be explained to a substantial degree by its features being more (or less) heavy tailed than expected. According to our argument, small cities that emulate the composition of big cities provide greater opportunities for cumulative advantage and thus enable tails to grow larger than expected. Supplementary Fig. 11 plots SATs against SAMIs for the simulation run calibrated to baseline parameters.

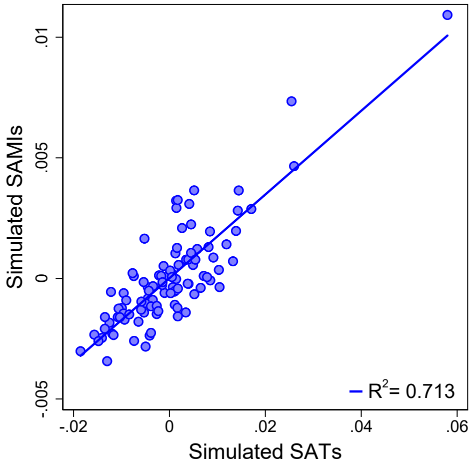

Supplementary Figure 11. Association between deviations in city tailedness and deviations from scaling predictions of urban output in the simulated data. Linear association between scale-adjusted tailedness (SATs) and scale-adjusted metropolitan indicators (SAMIs) [slope = 0.174 ± 0.021 (95% confidence interval around the coefficient obtained from linear regression), $R^2 = 0.713$].

Note 5. Empirical identification of cumulative advantage and selective migration as drivers of tail differences

To empirically investigate how cumulative advantage on the micro level translates into tail differences by city size on the macro level, we follow a two-step procedure. First, we estimate dynamic earnings functions separately for those initially earning median wages (40th–60th percentile) or tail wages (≥90th percentile) within each city. By estimating earnings functions separately for the two groups, we allow for differential wage trajectories depending on initial endowments, as expected from theories of cumulative advantage[12–14].

We estimate a Mincerian type of earnings equation that models the wage growth rate, $w_{i,t}$, for individual i over years t as a non-linear function of accumulated labor market experience[14,15]:

$$w_{i,t} = \gamma_1 e_{i,t} + \gamma_2 e_{i,t}^2 + \gamma_3 e_{i,t}^3 + \alpha_i + \epsilon_{i,t},$$

where e_i represents accumulated experience and takes the value 0 in the first year (age 30) and thereafter increases to 10 (age 40), α_i is an individual-level fixed effect absorbing earners' time-constant characteristics (e.g., cognitive ability, family background), and $\epsilon_{i,t}$ is a normally distributed error with zero mean. The coefficients γ_1, γ_2, and γ_3 capture the average first, second, and third order effects that changes in $e_{i,t}$ have on $w_{i,t}$, estimated based on variance within each individual's trajectory.

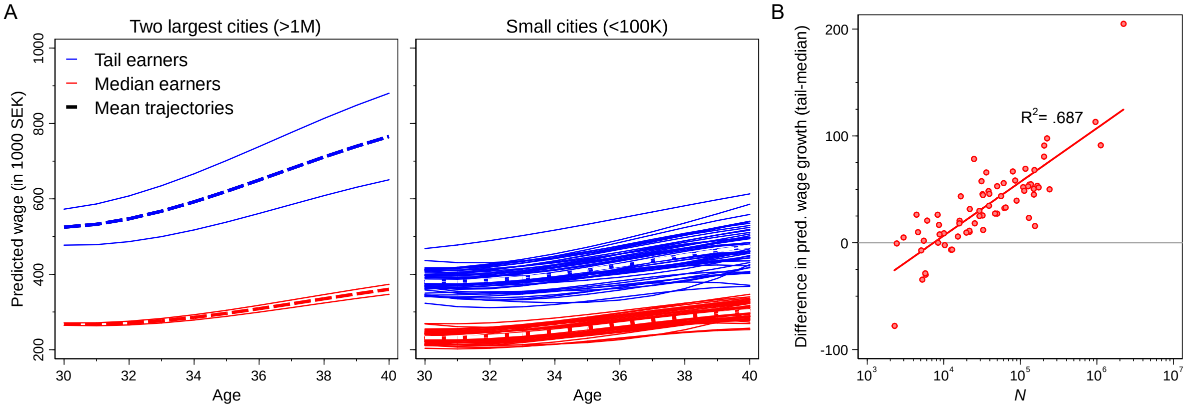

Supplementary Figure 12. The city size-dependent cumulative advantage effect. (**A**) Estimated wage growth of tail and median earners between ages 30 and 40 separately for Swedish labor market areas, based on the non-linear regression model that conditions individual wage growth on accumulated labor-market experience. We differentiate the two largest labor market areas Stockholm and Göteborg (left), and the 50 labor market areas with <100 thousand inhabitants (right), and we add—as intercepts—the city-specific average cohort wage at age 30. (**B**) In support of the predicted city size-dependent cumulative advantage effect, the wage-growth difference between tail earners and median earners increases by log. city size. The red line provides a linear approximation of this relationship [slope = 21.846±5.796 (95% confidence interval around the coefficient obtained from linear regression), $R^2 = 0.687$].

We trace the wage developments for all annual cohorts from 1990–2007 over 10 years. For all members of a cohort, we estimate the wage equation separately for each intersection of (*i*) city, (*ii*) wage percentile (median or tail), and (*iii*) education level (with or without college degree). As a way of summarizing the results of these models, Supplementary Fig. 12A plots

the predicted wage growth of median and tail earners between ages 30 and 40 separately for each city. We differentiate the two largest labor market areas (Stockholm and Göteborg) and the 50 labor market areas with <100 thousand inhabitants. Supplementary Fig. 12B shows the difference in predicted wage growth (tail – median) for each city. In confirmation of the predicted city size-dependent cumulative advantage effect, we observe that the difference in estimated wage growth between tail earners and median earners systematically increases by city size.

Second, we use the estimated earnings equations to measure the impact that the observed cumulative advantage effects have on tail differences. To do so, we estimate counterfactual wage trajectories wherein we cancel any cumulative advantage effects by letting initial tail earners only grow their wages at the rate of median earners (with education levels similar to those of the tail earners) in their respective cities. We then re-compute the between-city scaling coefficient β for each age of cohort members (blue squares in Fig. 4C). The inset in Fig. 4C shows that our estimated equivalents to the real-world wage trajectories reproduce the observed scaling exponents by cohort age.

Finally, to quantify the impact of selective migration on cities' tail differences, we update a cohort g's city-wide output at cohort age t (the numerator $Y_{g,t}$) to include the wages of those earners who have moved to these cities, while keeping the city-specific cohort size (the denominator $N_{g,t}$) fixed to the initial number of people in that city's cohort (dark blue line in Fig. 4C). The scaling exponent then reflects both emergent and selection-based output differences by city size. Supplementary Fig. 13 shows the extent of selective migration in Sweden, where—as mentioned in the main text—individuals classified as tail earners at age 30 are twice as likely to have left a small city compared to a large city until age 40, and those leaving their area overwhelmingly tended to move into the largest cities (inset).

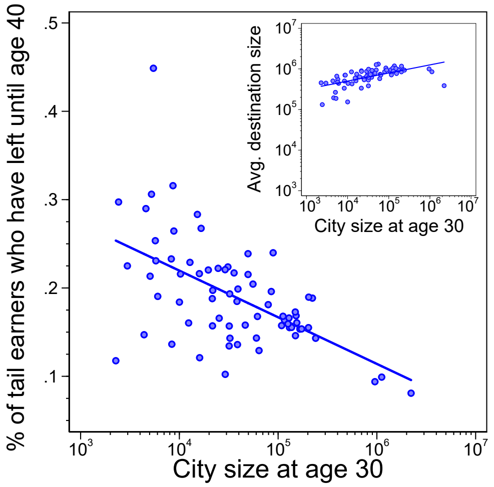

Supplementary Figure 13. The extent of selective migration. Focusing on tail earners (≥90th percentile within each city), we find them leaving smaller cities at higher rates than larger cities and tending to move into the largest cities (inset).

Note 6. Stochastic geometric growth and city tails as drivers of urban scaling

In a recently proposed framework[11], important steps have been taken in the direction of accounting for heterogeneity and inequality in urbanization processes. Here, we (*i*) review how inequality emerges in this framework, (*ii*) elaborate on our claim that the framework nonetheless either precludes or remains neutral to tails as *drivers* of urban scaling, and (*iii*) demonstrate how the type of mechanisms identified in our study can be incorporated in order to align the framework's predictions with the empirical observations that fine-grained micro-level data on city distributions provide.

The framework revolves around two central assumptions: (*i*) agents have a budget condition, $y - c$, where y is income and c is cost, and (*ii*) agents act strategically to control fluctuations in net incomes—which they do by the temporal averaging of expenditures. These assumptions are translated into a model of stochastic multiplicative growth

$$\frac{dr(t)}{dt} = y(t) - c(t) = \eta_r(t)r(t) \tag{1}$$

where $r(t)$ is the accumulated net quantity of $y(t)$ over time t, and η_r is the stochastic growth rate of r. Integrating its solution in time leads to

$$\log \frac{r(t)}{r(0)} = (\bar{\eta_r} - \frac{\sigma_r^2}{2})t + \Theta(t) \tag{2}$$

where $\bar{\eta_r}$ and σ_r^2 are the mean and variance of η_r, respectively, and $\Theta(t)$ is stochastic noise. Then, the assumption that η_r is statistically independent across time implies that $\log \frac{r(t)}{r(0)}$ approaches, in the limit of long times, a Gaussian variable with time-dependent mean $(\bar{\eta_r} - \frac{\sigma_r^2}{2})t$ and variance $\sigma_r^2 t$. This, in turn, implies that $r(t)$ is asymptotically distributed as a log-normal variable. The accumulation of stochastic growth rates over time represents the *first* and most central way in which within-city inequality emerges in the model.

The *second* way that within-city inequality can emerge follows from the manner in which agent-level behavior is aggregated. To derive the dynamics for the level of a city, the framework considers averages of each quantity—e.g., the aggregate resources for a city of size N is defined as $r_N = \frac{1}{N}\sum_i^N r_i$, where r_i are individual i's resources. Then, the temporal dynamics for city-wide resources is computed as

$$\frac{dr_N}{dt} = y_N - c_N = (\eta r)_N \tag{3}$$

where the product $(\eta r)_N$ is decomposed as

$$(\eta r)_N = \frac{1}{N}\sum_{i=1}^{N} \eta_i r_i = \eta_N r_N + covar_N(\eta, r) = [\eta_N + covar_N(\eta, r/r_N)]r_N \tag{4}$$

Here, the second source of inequality becomes clear: a positive covariance between η and r implies that agents with greater resources also exhibit greater growth rates, and vice versa.

What do these two sources of inequality imply for the emergence of tail differences by city size, and for tails as drivers of urban scaling? The first source—the accumulation of

stochastic growth rates over time—produces inequality within cities, but it does so to the same extent in cities of varying sizes. As a result, the tailedness of a city is presumed size-independent, and urban inequality has no implications for between-city scaling. For demonstration, we performed replicative simulations following the procedure of the original manuscript based on equation (2), and using the same parameters (derived from US MSAs: $\bar{\eta} = 0.06$, $\sigma = 0.045$; $\Theta(t) \sim N(0,1)$). We simulated resource trajectories for 100 fictive cities of different sizes (the largest having 1 million inhabitants, the others with decreasing sizes following Zipf's law), and we computed the 10:90-ratio d for each city. The inset of Supplementary Fig. 14A demonstrates the assumed independence of d and N. For these results, we varied the total number of iterations t in the simulation, and with it the length of the resulting cumulative advantage chains. Increasing t affects the average tailedness, $\bar{d}$, across artificial cities but it does not affect the difference in d by city size and, hence, has no effect on the degree of scaling, β.

With respect to the second source—the positive covariance between resources and growth rates—ref. 17 does not derive any city-level dynamics or system-level implications. Notably, from what is derived, no prediction is made regarding the relationship between covariance and city size. To assess the implications that covariance between resources and growth rates have on within-city tailedness and between-city scaling, we expanded on the simulation model by instantiating variation in agents' initial resources and adding covariance between $\bar{\eta}$ and $r(0)$. Specifically, for each agent i in city j, we assigned: $\bar{\eta}_{ij}, \frac{r_{ij}(0)}{r_j(0)} \sim \mathcal{N}\left(\boldsymbol{\mu} = (0.06, 1), \boldsymbol{\Sigma} = \begin{bmatrix} 0.01 & c \\ c & 0.1 \end{bmatrix}\right)$, where the ratio $r_{ij}(0)/r_j(0)$ reflects individual i's initial resources relative to the average of city j, and c is a constant that controls the magnitude of the covariance and thus moderates the strength of the cumulative advantage process. We varied c in different simulation runs, using the same value of c across cities for each run. Supplementary Fig. 14B shows that, with rising covariance c, cities' average tailedness $\bar{d}$ systematically increases. However, there is no difference in tailedness by city size and—as with the first source of inequality—the second source of inequality has no implications for between-city scaling.

These simulations shed light on a disconnect that exists between inequality and scaling in the proposed framework: what generates inequality does not affect scaling. By decoupling inequality from scaling, the referenced paper implicitly reinforces the traditional perspective on urban scaling as being driven by differences in the mass of cities rather than their tails.* However, by incorporating the city size-dependent cumulative advantage mechanism identified in our study, the framework's predicted dynamics can be brought into accordance with the empirical observations we reported in Figs. 2 and 3. A natural way of implementing size-dependent cumulative advantage would be to instantiate a positive dependence between the covariance of resources and growth rates, c, and city size N. Correspondingly, we extended the simulation procedure underlying Supplementary Fig. 14B by assigning city-

*The framework's alignment with the traditional mass-shift perspective is further strengthened by the fact that the derived conditions under which scaling is not preserved over time are reliant on a particular mechanism—size-dependent growth-rate volatility σ^2—which reduces the fluctuations of growth rates in larger as compared to smaller cities and, as a result, homogenizes growth, reduces inequality, and shifts the mass upwards in larger cities.

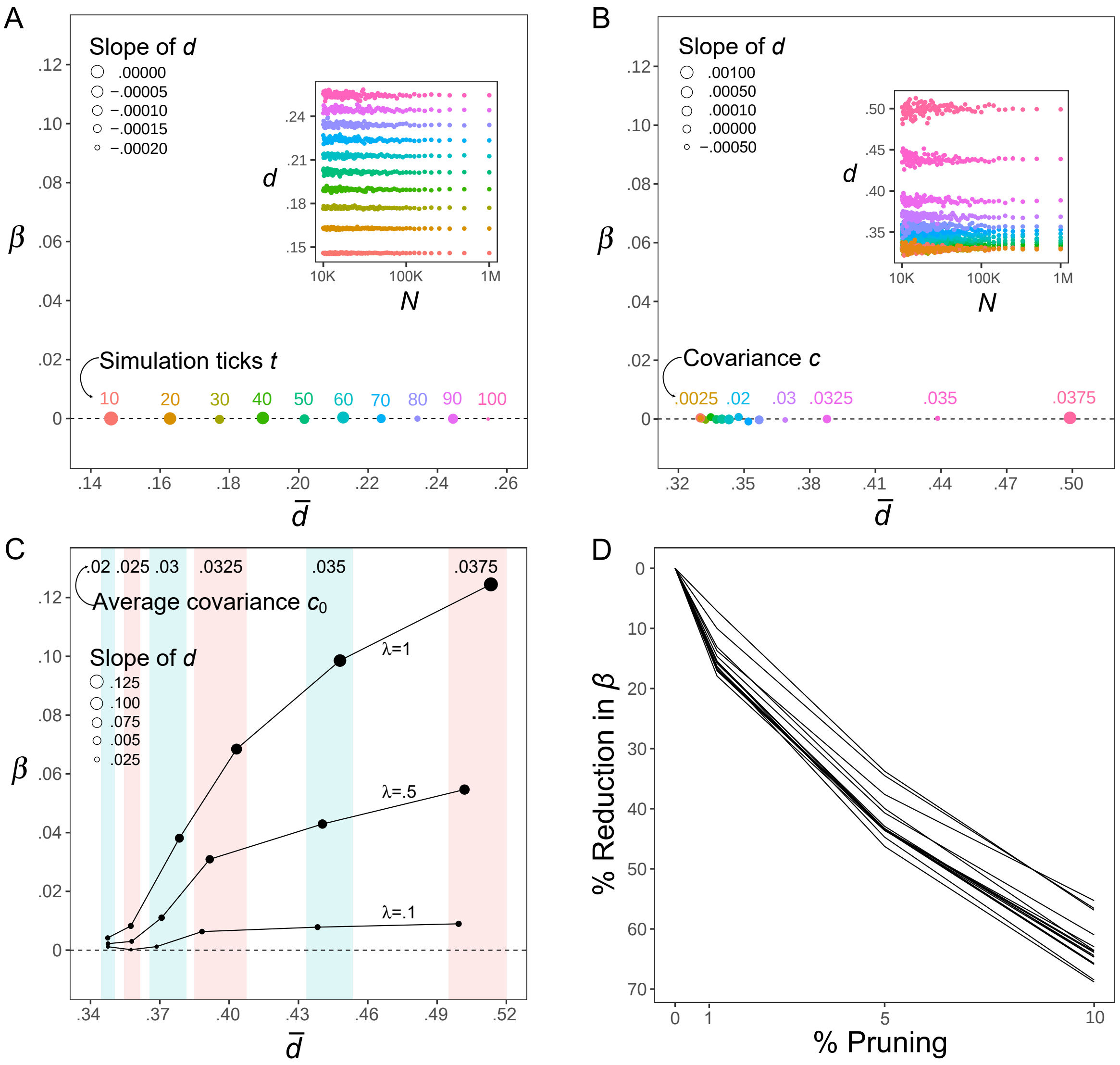

Supplementary Figure 14. Replicative simulations highlight the disconnect between inequality and scaling in ref. 17 (panels A and B), but they also show how the incorporation of a size-dependent cumulative advantage mechanism aligns the predictions of the framework with the empirical observations reported here (panels C and D). (**A**) The first simulation scenario evaluates the accumulation of stochastic growth rates over time and varies the length, t, of the simulation. Increasing t boosts cities' average tailedness, $\bar{d}$, but does not affect the difference in d by city size (slope of d), and thus has no effect on β. (**B**) The second scenario varies the covariance, c, between resources and growth rates. As a result, $\bar{d}$ increases with rising c, but there is still no difference in tailedness by city size and no effect on β. The insets demonstrate the assumed independence of d and N in ref. 17. (**C** and **D**) Incorporating the city size-dependent cumulative advantage mechanism identified in our study brings the predicted dynamics of ref. 17 into accordance with our empirical observations (cf., Figs. 2 and 3). The third scenario varies cities' average covariance, c_0, and its size-dependency, λ. Higher values on both parameters increase $\bar{d}$, the slope of d, and β, reproducing the key characteristics of our empirical results.

specific covariance terms $c_j = c_0 \left(\log(N_j)^\lambda / (\frac{1}{K}\sum_{j=1}^{K} \log(N_j)^\lambda)\right)$, where c_0 controls the average covariance across cities, and λ moderates the strength of the city-size dependence on the covariance. Supplementary Fig. 14C shows that—with rising c_0 and rising λ—both $\bar{d}$ and the slope of d, as well as β increase systematically, reproducing the key characteristics of our

empirical results (see also Supplementary Fig. 14D).[†] In other words, implementing our proposed mechanism in this other existing framework produces the same insights as obtained with our computational model, demonstrating the generalizability and explanatory power of the mechanism.

[†]The influence that the cumulative advantage mechanism proposed here has on the preservation of scaling over time depends on the mechanism's particular instantiation. If one were to take a life-course perspective, for example, there would be a continuous in- and outflow of labor-market participants as a function of individuals moving through different life stages (e.g., education-to-work transition, retirement). Individuals' relative positions in terms of resources would then be defined in relation to the members of their cohort, and both within-city inequality and between-city scaling would—as demonstrated in Fig. 4—emerge over the life course. Scaling would then be preserved over time if there were no inter-generational dependence.

Note 7. Interconnectivity depends on the extremely interlinked

In addition to assuming that individuals within a city have similar levels of interconnectivity—as captured by network degree—urban scaling research typically also assumes strong levels of stuctural homogeneity, conceiving of network nodes as having very similar types of roles or positions. However, as the literature on network diffusion demonstrates[16–18], in real-world networks some nodes are much more important than others for the spread of information and for coordination processes. Networks contain higher-order dependencies such that the removal of any given node not only affects its direct neighbors, but also more distant nodes through the removal of particular channels of network flows.

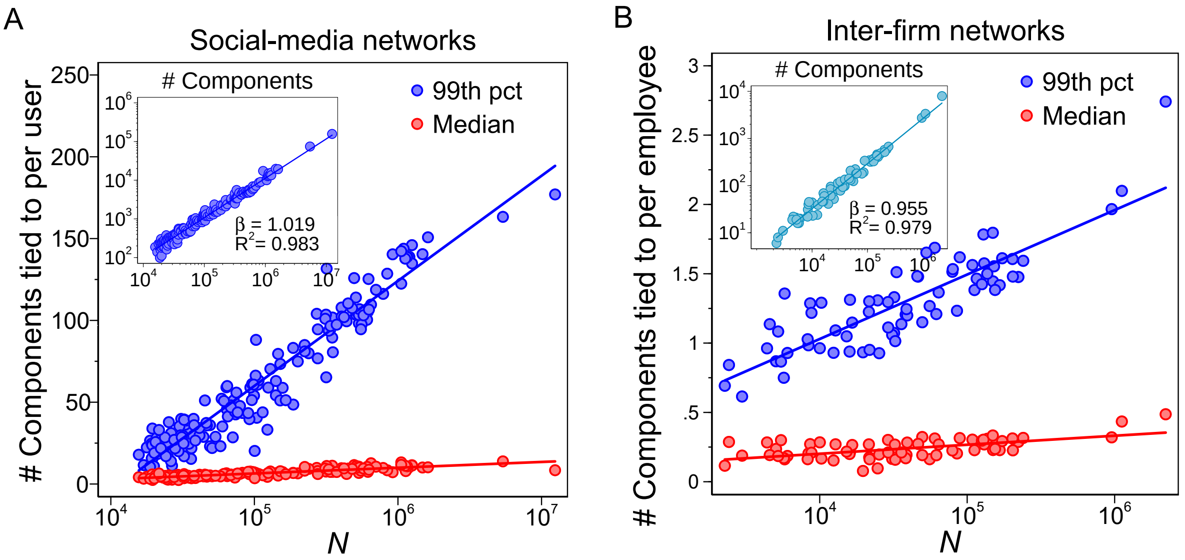

Supplementary Figure 15. Interconnectivity depends on the extremely interlinked as cities become larger. Networks in larger cities are increasingly fractured, encompassing greater numbers of components in both social media networks and inter-firm networks (insets). (**A**) Average number of network components to which social media users connect, by city size. We use the community detection algorithm InfoMap[19] to partition the networks. Nodes with a degree in the 99th percentile (dark blue) connect to more components in a city's network as log. city size increases [slope $= 27.882 \pm 1.145, R^2 = 0.924$]. Cities' typical nodes (red), by contrast, connect to relatively few components, and the number of components remains largely unaffected by city size [slope $= 1.527 \pm 0.201, R^2 = 0.759$]. (**B**) Similar results hold for inter-firm networks in the Swedish labor market areas. Firms scoring a top-1% degree connect, per employee, to more network components (e.g., specialized industries) as city sizes increase [dark blue: slope $= 0.202 \pm 0.042, R^2 = 0.731$], while this association is almost flat for the median firm [red: slope $= 0.028 \pm 0.012, R^2 = 0.341$].

Our micro-level data reveal that interconnectivity not only depends on the extremely interlinked via their immediate or first-order connectivity (as measured by their degree; see "interconnectivity" in Fig. 2B), but also indirectly via their structural positions in the networks—and that this higher-order dependency increases with city size. As cities become larger, their networks become more small-world-like, exhibiting increased fragmentation while retaining short average path distances (Supplementary Fig. 15 insets). Supplementary Fig. 15 examines, for the social media network and the inter-firm network, the average number of components interconnected through specific node types in cities of varying sizes. While the typical social media user and the typical firm (standardized by the number of employees) is tied to a similar number of network clusters in smaller and larger cities, the tail

nodes of these networks are tied to an increasing number of network clusters as city size increases, reflecting their increasing importance as bridges between clusters as one moves from smaller to larger cities. These results suggest that, in increasingly fragmented urban networks, the extremely interlinked act as important bridges facilitating flows between distant parts of the network and the effective combination of distributed specializations.

References

1. Keuschnigg, M., Mutgan, S. & Hedström, P. Urban scaling and the regional divide. *Sci. Adv.* **5,** eaav0042 (2019).
2. Ganzeboom, H. B., De Graaf, P. M. & Treiman, D. J. A standard international socio-economic index of occupational status. *Soc. Sci. Res.* **21,** 1–56 (1992).
3. Bettencourt, L. M. A., Lobo, J., Helbing, D., Kühnert, C. & West, G. B. Growth, innovation, scaling, and the pace of life in cities. *Proc. Natl. Acad. Sci. U. S. A.* **104,** 7301–6 (2007).
4. Schläpfer, M. *et al.* The scaling of human interactions with city size. *J. R. Soc. Interface* **11,** 20130789 (2014).
5. Ortman, S. G., Cabaniss, A. H. F., Sturm, J. O. & Bettencourt, L. M. A. Settlement scaling and increasing returns in an ancient society. *Sci. Adv.* **1,** e1400066 (2015).
6. Bettencourt, L. M. A. The origins of scaling in cities. *Science* **340,** 1438–41 (2013).
7. Shalizi, C. R. Scaling and hierarchy in urban economies. *arXiv,* 1102.4101 (2011).
8. Bettencourt, L. M. A., Lobo, J., Strumsky, D. & West, G. Urban scaling and its deviations: Revealing the structure of wealth, innovation and crime across cities. *PloS ONE* **5,** e13541 (2010).
9. Neffke, F. M. H. The value of complementary co-workers. *Sci. Adv.* **5,** eaax3370 (2019).
10. Adamou, A. & Peters, O. Dynamics of inequality. *Significance* **13,** 32–35 (2016).
11. Bettencourt, L. M. A. Urban growth and the emergent statistics of cities. *Sci. Adv.* **6,** eaat8812 (2020).
12. Merton, R. K. The Matthew effect in science. *Science* **159,** 56–63 (1968).
13. MacDonald, G. M. The economics of rising stars. *Am. Econ. Rev.* **78,** 155–166 (1988).
14. DiPrete, T. A. & Eirich, G. M. Cumulative advantage as a mechanism for inequality. *Annu. Rev. Sociol.* **32,** 271–297 (2006).
15. Mincer, J. *Schooling, Experience, and Earnings* (Columbia Univ. Press, 1974).
16. Granovetter, M. The strength of weak ties. *Am. J. Sociol.* **78,** 1360–80 (1973).
17. Burt, R. S. *Structural Holes* (Harvard Univ. Press, 1992).
18. Watts, D. J. & Strogatz, S. H. Collective dynamics of 'small-world' networks. *Nature* **393,** 440–442 (1998).
19. Rosvall, M. & Bergstrom, C. T. Maps of random walks on complex networks reveal community structure. *Proc. Natl. Acad. Sci. U. S. A.* **105,** 1118–23 (2008).
