# Supplementary material for: Urban scaling laws arise from within-city inequalities
Source: Nat Hum Behav. 2023 Jan 26;7(3):365–74. doi: 10.1038/s41562-022-01509-1 (PMC10038794; doi:10.1038/s41562-022-01509-1)
Supplement: Supplementary file 2 — Reporting Summary [file 41562_2022_1509_MOESM2_ESM.pdf]

nature research

Corresponding author(s): Martin Arvidsson, Niclas Lovsjö, Marc Keuschnigg

Last updated by author(s): November 24, 2022

Reporting Summary

Nature Research wishes to improve the reproducibility of the work that we publish. This form provides structure for consistency and transparency in reporting. For further information on Nature Research policies, see our Editorial Policies and the Editorial Policy Checklist.

Statistics

For all statistical analyses, confirm that the following items are present in the figure legend, table legend, main text, or Methods section.

n/a	Confirmed	
☐	☒	The exact sample size (*n*) for each experimental group/condition, given as a discrete number and unit of measurement
☐	☒	A statement on whether measurements were taken from distinct samples or whether the same sample was measured repeatedly
☐	☒	The statistical test(s) used AND whether they are one- or two-sided<br>*Only common tests should be described solely by name; describe more complex techniques in the Methods section.*
☐	☒	A description of all covariates tested
☐	☒	A description of any assumptions or corrections, such as tests of normality and adjustment for multiple comparisons
☐	☒	A full description of the statistical parameters including central tendency (e.g. means) or other basic estimates (e.g. regression coefficient) AND variation (e.g. standard deviation) or associated estimates of uncertainty (e.g. confidence intervals)
☐	☒	For null hypothesis testing, the test statistic (e.g. *F*, *t*, *r*) with confidence intervals, effect sizes, degrees of freedom and *P* value noted<br>*Give P values as exact values whenever suitable.*
☒	☐	For Bayesian analysis, information on the choice of priors and Markov chain Monte Carlo settings
☐	☒	For hierarchical and complex designs, identification of the appropriate level for tests and full reporting of outcomes
☐	☒	Estimates of effect sizes (e.g. Cohen's *d*, Pearson's *r*), indicating how they were calculated

Our web collection on statistics for biologists contains articles on many of the points above.

Software and code

Policy information about availability of computer code

Data collection	We used R [Version 4.0.5] to collect the online networking data and the data on patents and research grants. Code is available upon request. Company and wage data were provided to us by Statistics Sweden.
Data analysis	We used R [Version 4.0.5] for data analysis and simulations, Python [Version 3.6.4] for InfoMap-based network clustering, and Stata [Version 15] for visualizations. Code is available for download at the Open Science Framework (DOI: 10.17605/osf.io/uhsmz; https://osf.io/uhsmz/)

For manuscripts utilizing custom algorithms or software that are central to the research but not yet described in published literature, software must be made available to editors and reviewers. We strongly encourage code deposition in a community repository (e.g. GitHub). See the Nature Research guidelines for submitting code & software for further information.

Data

Policy information about availability of data

All manuscripts must include a data availability statement. This statement should provide the following information, where applicable:

- Accession codes, unique identifiers, or web links for publicly available datasets
- A list of figures that have associated raw data
- A description of any restrictions on data availability

The data that support the findings of this study are available for download at the Open Science Framework (DOI: 10.17605/osf.io/uhsmz; https://osf.io/uhsmz/). We collected the online networking data for Russia and Ukraine through the VKontakte API (https://vk.com/dev/openapi), the data on US patents are from the US Patent and Trademark Office (https://www.patentsview.org) and on research grants from Dimensions (https://www.dimensions.ai). The Swedish micro-level data come from administrative and tax records and can therefore not be shared; access may be requested from Statistics Sweden (https://scb.se/en/services/guidance-for-researchers-and-universities). We use publicly available data on city demarcations and population sizes from the Russian Federal State Statistics Service (https://

eng.gks.ru), the State Statistics Committee of Ukraine (http://ukrstat.gov.ua), Statistics Sweden (https://scb.se/en), and the United States Census Bureau (https://www.census.gov).

Field-specific reporting

Please select the one below that is the best fit for your research. If you are not sure, read the appropriate sections before making your selection.

☐ Life sciences ☒ Behavioural & social sciences ☐ Ecological, evolutionary & environmental sciences

For a reference copy of the document with all sections, see nature.com/documents/nr-reporting-summary-flat.pdf

Behavioural & social sciences study design

All studies must disclose on these points even when the disclosure is negative.

Study description	The study analyzes quantitative micro-level data aggregated to the city-level in urban systems in Europe and the United States. The study demonstrates how urban scaling laws arise from within-city inequality. We show that indicators of interconnectivity, productivity, and innovation have heavy tailed distributions in cities, and that city tails, and their growth with city size, play an important role in the emergence of urban scaling. With agent-based simulation and an analysis of longitudinal micro-level data, we identify a city-size dependent cumulative advantage mechanism behind differences in the tailedness of urban indicators by city size.
Research sample	Our datasets are based on a complete sampling of the relevant populations. For Sweden, the data include all fully employed earners in 2017 (3.4 million) and all private companies during 2015-17 (260 thousand) in Sweden's 70 labor market areas. The longitudinal data on earning trajectories includes all 1.4 million earners between 1990 and 2017. The data on online networking includes all private user accounts at VKontakte in Russia (40 million in 177 cities) and Ukraine (10.5 million in 87 cities) in 2018 that have >1 and < 5,000 contacts and that were registered in a city where >=1/3 of the population have VKontakte accounts (see Supplementary Note 1). The US patent data cover 8.3 million patents from 1.8 million inventors registered during 1976-2019 in 379 Metropolitan Statistical Areas (MSAs). The US grant data cover 892 thousand grants awarded to 363 thousand researchers during 1960-2019 in 218 MSAs. In each dataset, full sampling of the relevant population ensures representativity of the underlying population.
Sampling strategy	In each dataset, we use a full census of the relevant population, hence no sampling strategy was applied.
Data collection	The data on online networking (https://vk.com/dev/openapi), patents (https://www.patentsview.org), and research grants (https://www.dimensions.ai) were publicly available on the web at the time of data collection. Data on companies (turnover and mobility) and individuals' wages were provided to us by Statistics Sweden.
Timing	Collection of online networking data, patents data, and grants data began in August 2019 and ended in May 2020. Statistics Sweden records data in November each year, and the last year we have data for is 2017.
Data exclusions	No data were excluded from the analysis.
Non-participation	Our use of process-produced and administrative data inhibits non-participation.
Randomization	Our conclusions do not rest on randomization and our observational design inhibits randomization.

Reporting for specific materials, systems and methods

We require information from authors about some types of materials, experimental systems and methods used in many studies. Here, indicate whether each material, system or method listed is relevant to your study. If you are not sure if a list item applies to your research, read the appropriate section before selecting a response.

Materials & experimental systems

n/a	Involved in the study	
☒	☐	Antibodies
☒	☐	Eukaryotic cell lines
☒	☐	Palaeontology and archaeology
☒	☐	Animals and other organisms
☒	☐	Human research participants
☒	☐	Clinical data
☒	☐	Dual use research of concern

Methods

n/a	Involved in the study	
☒	☐	ChIP-seq
☒	☐	Flow cytometry
☒	☐	MRI-based neuroimaging
